# Social isolation and poor mental health in young people: testing genetic and environmental influences in a longitudinal cohort study

**DOI:** 10.1007/s00787-024-02573-w

**Published:** 2024-09-11

**Authors:** Katherine N. Thompson, Olakunle Oginni, Jasmin Wertz, Andrea Danese, Malaika Okundi, Louise Arseneault, Timothy Matthews

**Affiliations:** 1 Department of Sociology, College of Liberal Arts, Purdue University, West Lafayette, IN United States of America; 2 Social, Genetic and Developmental Psychiatry Centre, Institute of Psychiatry, Psychology and Neuroscience, King’s College London, London, UK; 3 Department of Mental Health, Obafemi Awolowo University, Ile-Ife, Nigeria; 4 Department of Psychology, School of Philosophy, Psychology & Language Sciences, University of Edinburgh, Edinburgh, UK; 5 Department of Child & Adolescent Psychiatry, Institute of Psychiatry, Psychology & Neuroscience, King’s College London, London, UK; 6 National and Specialist CAMHS Trauma, Anxiety, and Depression Clinic, South London and Maudsley NHS Foundation Trust, London, UK; 7 School of Human Sciences, Faculty of Education, Health and Human Sciences, University of Greenwich, London, UK

**Keywords:** Social isolation, Genetic overlap, Longitudinal, Depression symptoms, Psychotic-like experiences, Conduct problems

## Abstract

We assessed genetic and environmental influences on social isolation across childhood and the overlap between social isolation and mental health symptoms including depression symptoms, conduct problems, and psychotic-like experiences from adolescence to young adulthood. Participants included 2,232 children from the Environmental Risk Longitudinal Twin Study. Social isolation was measured at ages 5, 7, 10, 12, and 18. A Cholesky decomposition was specified to estimate the genetic and environmental influences on social isolation across ages 5, 7, 10, and 12. An independent pathway model was used to assess additive genetic (A), shared environmental (C), and non-shared environmental (E) influences on the overlap between social isolation and mental health problems from age 12 to 18. Genetic and non-shared environmental influences accounted for half of the variance in childhood social isolation. Genetic influences contributed to the continuity of social isolation across childhood, while non-shared environmental influences were age-specific. The longitudinal overlap between social isolation and mental health symptoms was largely explained by genetic influences for depression symptoms (*r =* 0.15–0.24: 82–84% A, 11–12% C, and 5–6% E) and psychotic-like experiences (*r =* 0.13–0.15: 81–91% A, 0–8% C, and 9–11% E) but not conduct problems (*r =* 0.13–0.16; 0–42% A, 42–81% C, 16–24% E). Our findings emphasise that rather than a risk factor or an outcome, social isolation is aetiologically intertwined with the experience of poor mental health. An integrative assessment of social isolation could be a helpful indicator of underlying mental health symptoms in young people.

**Supplementary Information:**

The online version contains supplementary material available at 10.1007/s00787-024-02573-w.

## Introduction

Social isolation is an established risk factor for poor mental health [1], although rarely considered in young people. The U.S. Surgeon General recently highlighted the importance of social isolation as a public health priority [2]. The potential impact of isolation on mental health could be particularly apparent in the transition from childhood to adulthood, as this is a crucial developmental period marked by biological, social, and psychological changes [3]. Relationships provide a buffer for navigating difficult experiences through companionship, guidance, and support in times of stress [4]. Increased social connectedness demonstrates promise for reducing the burden of mental health disorders in young people [4].

Socially isolated individuals could be at increased risk for depression, anxiety, suicidality, cardiovascular problems, obesity, inflammation, accelerated cognitive decline, low educational attainment, and premature mortality [1, 5–7]. Being socially isolated from other people is distinct from feeling lonely and has shown unique associations with mental health problems [8]. This link is complex, with a range of studies reporting concurrent, longitudinal, and bidirectional associations [7–11]. Whilst social isolation has been conceptualised as a risk factor for poor mental health, isolation can also occur as a consequence of experiencing childhood psychiatric symptoms [9, 10]. Individuals with mental health disorders, such as depression, conduct disorder, and psychosis consistently report social difficulties alongside their core symptoms [12–14].

The co-occurrence of social isolation and mental health disorders is multifactorial. Similar to other social behaviours [15–17], social isolation is ~ 40% heritable in adulthood [18]. This genetic predisposition suggests a gene-environment correlation (*r*GE), whereby genetic influences can shape the environment an individual is situated in. An “environmental” exposure, such as social isolation, can be partly explained by genetic differences. Being isolated arises from an individual’s surroundings, but how an individual responds to that environment reflects a heritable component [19]. Genetic influences on social isolation can arise, for example, if genetically-influenced characteristics make it more likely that someone becomes isolated. The heritability of social isolation can differ in childhood or adulthood for two key reasons. First, the phenotype can present differently. Isolation in adulthood is an absence of any relationships [16]. Children are typically surrounded by parents and siblings; therefore, isolation represents a lack of connections with peers [5]. This approach captures developmental changes in genetic architecture that reflect the variation in how children form friendships over time. Second, genetic and environmental influences could change over time as social behaviour transforms throughout development [20]. As children grow up, relationships become intricate and demanding, and peer interactions are vital to development [3]. Identifying periods when environmental experiences have the strongest impact could provide avenues for timely interventions.

A common underlying genetic liability could contribute to the co-occurrence of social isolation and mental health problems [18]; the same heritable characteristics involved in mental health disorders could drive individuals to be isolated from social groups. Twin and polygenic methods have reported genetic overlap between various forms of social behaviour and mental health symptoms [18, 21–23]. Genetic influences contribute to initial experiences and developmental continuity in behaviour whereas environmental factors tend to explain behavioural or symptom changes over time [24]. A better understanding of the genetic and environmental influences on the overlap between social isolation and mental health disorders across development can help us understand the dynamic aetiological processes and provide insight into the directionality of this association.

Using data from a nationally representative longitudinal United Kingdom cohort, we (a) assessed the genetic and environmental influences on social isolation from age 5 to 12 and (b) explored the genetic and environmental influences on the longitudinal overlap between social isolation and mental health symptoms including depression symptoms, conduct problems, and psychotic-like experiences across ages 12 to 18.

## Methods

### Participants

Participants were members of the Environmental Risk (E-Risk) Longitudinal Twin Study, which tracks the development of 2,232 British children. The sample was drawn from a larger birth cohort of twins born in England and Wales in 1994–1995. Full details are reported elsewhere [25]. E-Risk was constructed in 1999–2000, when 1,116 families (93% eligible) with same-sex 5-year-old twins participated in home-visit assessments. This sample comprised 56% monozygotic (MZ) and 44% dizygotic (DZ) twin pairs; sex was evenly distributed within zygosity (49% male); 90% of participants were of White ethnicities. The sample represents socioeconomic conditions in the UK, as reflected in the families’ distribution on neighbourhood-level socioeconomic indices. Follow-up home visits were conducted when children were aged 7 (98% participation), 10 (96%), 12 (96%), and 18 years (93%). Visits at ages 5–12 included assessments with participants and their mother (primary caretaker). The home visit at age 18 included interviews only with the participants. The Joint South London and Maudsley and the Institute of Psychiatry Research Ethics Committee approved each phase of the study. Between age 5–12, parents gave informed consent and participants gave assent, and participants then gave informed consent at age 18.

### Measures

Measures of social isolation, depression symptoms, conduct problems, and psychotic-like experiences are described in detail in Supplement 1 A. Briefly, social isolation was measured using the Child Behaviour Checklist and Teacher Report Form at ages 5, 7, 10, and 12, and the Multidimensional Scale of Perceived Social Support at age 18. Depression was measured using the Children’s Depression Inventory at age 12 and a structured diagnostic interview at age 18. Conduct problems were measured using a computerised questionnaire of antisocial behaviours based on DSM-IV criteria for conduct disorder at age 12 and 18. Psychotic-like experiences were measured using a structured interview on experiences of hallucinations and delusions at age 12 and 18.

### Statistical analyses

We used statistical methods based on the classical twin design [26] to test the influence of genetic and environmental factors on social isolation and mental health symptoms. MZ twins share 100% and DZ twins share approximately 50% of their genes on average. Through comparing the within-pair similarity on a trait in MZ versus DZ twins, we can estimate the influence of additive genetic (A), shared environmental (C), and non-shared environmental (E) factors on that trait. Additive genetic influences reflect the sum total effect of all genetic variants, which make twins more similar. The shared environment represents experiences that make twins similar, whereas non-shared environmental influences are unique to each twin and contribute to differences between twins while incorporating measurement error. All models were fitted using raw data maximum likelihood. Variables were regressed for sex and normalised with the most optimum transformation. Analyses were conducted using OpenMx [27] in R (4.0.3) and pre-registered (https://sites.duke.edu/moffittcaspiprojects/files/2022/09/ThompsonK_2022b_Social_Isoln_mental-health_genetic_overlap.pdf*).* All code is available on GitHub: http://github.com/knthompson26/isolation_mentalhealth_overlap.

We specified univariate ACE models to provide initial estimates of genetic and environmental influences and to test for quantitative and scalar (variance) sex differences on each trait (social isolation, depression symptoms, conduct problems, and psychotic experiences) at ages 12 and 18. To test for genetic and environmental influences on social isolation across childhood, we specified a Cholesky decomposition incorporating the measurements at ages 5, 7, 10, and 12 years. 𝝌^2^ difference tests were used to assess if a more parsimonious AE model fit the data better than the corresponding ACE model. To assess genetic and environmental influences on the longitudinal overlap between social isolation and mental health symptoms between ages 12 and 18 (depression symptoms, conduct problems, and psychotic experiences), we fit an independent pathway model (IPM). The IPM separated ACE influences into those *common* to all variables (A_C1_, C_C1_, and E_C1_) at both ages 12 and 18, those *common* to the variables at only age 18 (A_C2_, C_C2_, and E_C2_), and those *specific* to variables at each time point (A_S1−8_, C_S1−8_, and E_S1−8_) [28]. There were scalar sex differences (sex differences in phenotypic variance) for all variables apart from depression at age 12 and scalar models were fitted in the IPM to account for this (Supplement 3B). There were quantitative sex differences for conduct problems at age 12, thus, separate IPM path estimates were specified for males and females. Small (< 0.05) non-significant parameter estimates were constrained to zero to derive a parsimonious model.

## Results

### Genetic and environmental influences on social isolation throughout childhood

**Fig. 1:**
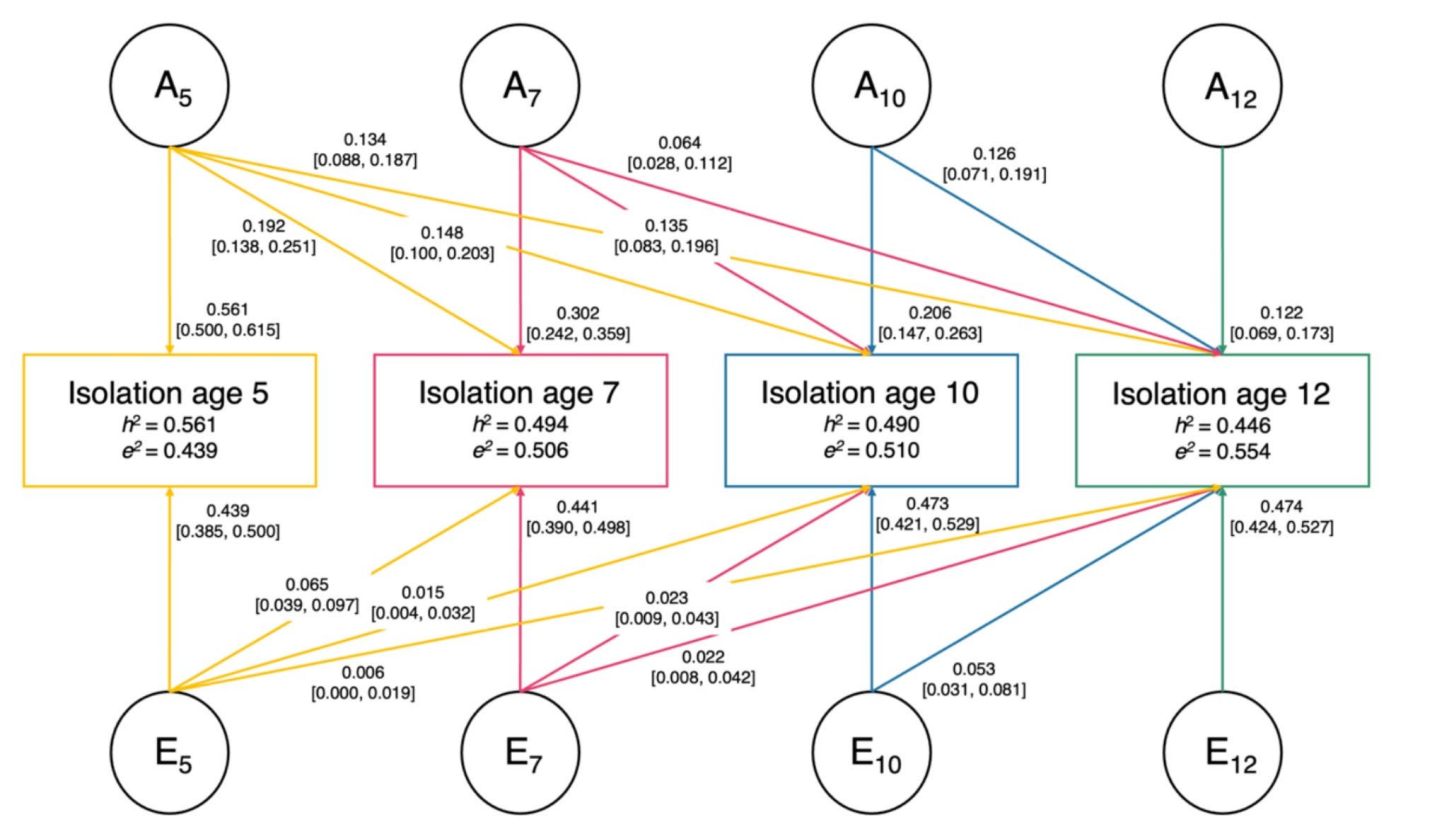
Cholesky decomposition of the additive genetic **(A)** and non-shared environmental **(E)** influences on social isolation at ages 5 (yellow), 7 (pink), 10 (blue), and 12 (green). Solid paths with single-headed arrows represent their (A and E) effects on isolation at the different time points. Path estimates indicate standardised contributions of each A and/or E component to social isolation at the different ages (subscript) and their 95% confidence intervals. For example, A_5_ explains 19.2% of the variance in social isolation at age 7. h^2^: heritability estimates, which represents the sum of standardised genetic influences from the previous and current time points. For example, h^2^ of social isolation at age 12 = 0.134 + 0.064 + 0.126 + 0.122 = 0.446 = 44.6% heritable, ~ 73% of which was in place before age 12 (0.134 + 0.064 + 0.126 = 0.324/0.446 = 72.64%)

When analysing genetic and environmental influences on social isolation at one point in time, we found that half the variance in childhood social isolation was accounted for by genetic influences (A; 56 − 45%) and half by non-shared environmental influences (E; 44–55%; Fig. 1; Supplement 2). There were negligible shared environmental (C) influences which were omitted from the model without significant loss in fit (Δ-2LL = 0.006, Δdf = 10, *p* = 0.999). When analysing genetic and environmental influences across time, our findings indicate that genetic factors contribute to initial levels and continuation of social isolation, while the non-shared environment impacts developmental or age-specific changes. We found that approximately 73% of the genetic influences on social isolation at age 12 emerged between ages 5 and 10 and 30% were already in place by age 5. New genetic effects emerged throughout development, but these substantially decreased as children aged (0.30 at age 7 to 0.12 at age 12). As opposed to genetic influences which contributed to continuity of social isolation, there were substantial distinct non-shared environmental influences (E) at each age (0.44–0.47) and few carry-over effects (0.01–0.07). Therefore, non-shared environmental influences were specific to each age, for example, only 14.6% of the variance in social isolation at age 12 was attributable to earlier experiences.

### Genetic and environmental influences on the overlap between social isolation and mental health symptoms from age 12 to 18

**Fig. 2:**
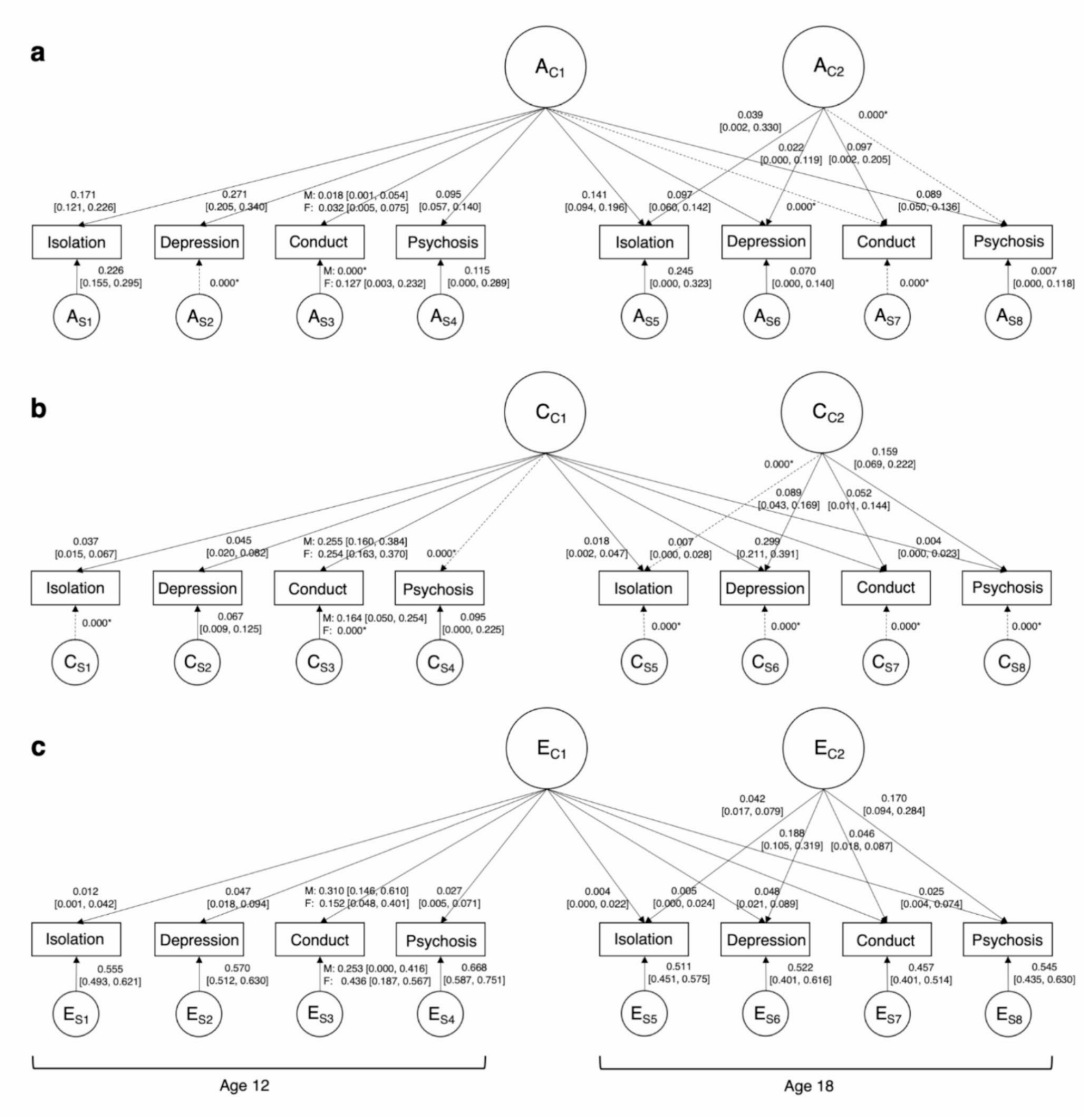
Independent pathway model (IPM) across ages 12 to 18. *A =* additive genetic influences, *C* = shared environmental influences, *E* = non-shared environmental influences on social isolation, depression symptoms, conduct problems, and psychotic experiences. Subscript C1 denotes common influences shared between traits and across time. Subscript C2 denotes common influences shared between traits at age 18. Subscript S1-8 denotes time- and trait-specific, residual influences. Solid paths denote A, C, and E influences on the variables at ages 12 and 18; the numbers on each path indicate their standardised contributions and their 95% confidence intervals. For example, A_C1_ and A_S_ explain 17.1% and 22.6% of the variance of social isolation at age 12 respectively, thus, 17.1% of the variance in social isolation at age 12 is explained by additive genetic factors common across all the other variables at ages 12 and 18, while 22.6% of this variance is explained by additive genetic influences not shared with any other variables. Dotted paths and * notation indicates paths that were constrained to be zero

### Concurrent associations

**Fig. 3:**
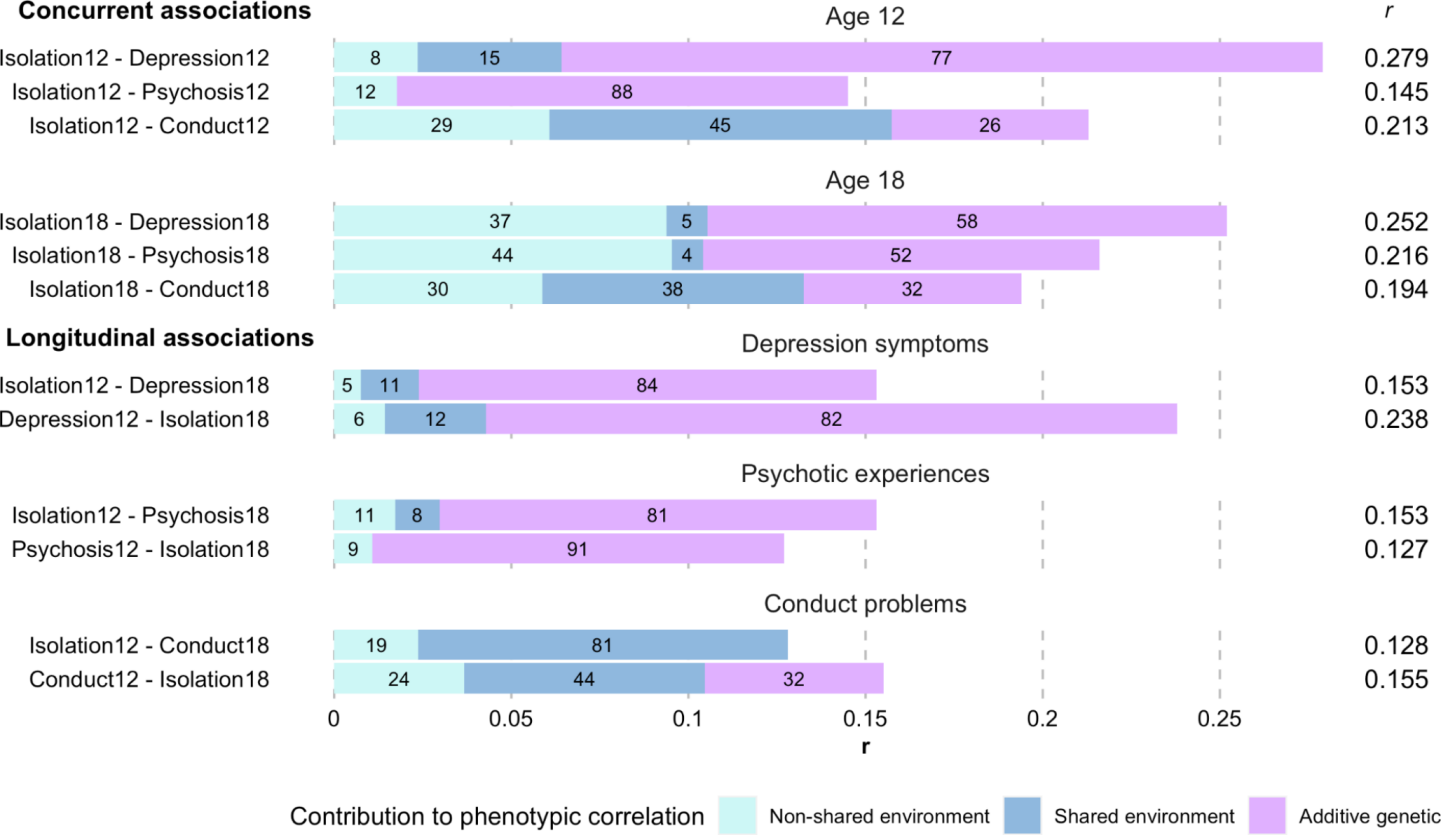
Proportions of concurrent and longitudinal associations between social isolation and mental health problems that are due to additive genetic **(A)**, shared environmental **(C)**, and non-shared environmental **(E)** influences. The size of the bars reflects the correlation coefficient (*r*) shown on the right hand side. The percentage of the phenotypic correlation attributed to A, C, and E are provided inside the bars. Percentages are estimated using path tracing rules applied to the model in Fig. 2. For example, the percentage of the correlation between social isolation age 12 and depression symptoms age 12 is calculated by multiplying the square root of all connecting paths, thus, √(0.171*0.271)/0.279 = 77.16%= 77% rounded. Estimates for conduct problems at age 12 are shown for *male participants only*. For *females*, contributions were: Isolation12 to Conduct12 (A = 35%, C = 45%, E = 20%) and Conduct12 to Isolation18 (A = 42%, C = 42%, E = 16%; see Supplement 3 C)

Concurrent phenotypic associations with social isolation were modest for all mental health symptoms (*r* = 0.15–0.28) and highest for depression symptoms both at age 12 (*r* = 0.28) and 18 (*r* = 0.25; Supplement 3 C). Genetic factors played a substantial explanatory role in concurrent associations between social isolation and both depression symptoms and psychotic experiences at age 12 (Figs. 2 and 3). When individuals reached age 18, genetic effects were reduced giving way to the non-shared environment that contributed three times more to co-occurrence of social isolation and mental health symptoms compared to age 12. Aetiological influences on concurrent associations between social isolation and conduct problems were consistent across ages 12 to 18; with nearly equal proportions of genetic, shared environmental, and non-shared environmental influences.

### Longitudinal associations

Longitudinal phenotypic associations were modest for all mental health symptoms (*r* = 0.13–0.24) and highest for depression symptoms at age 12 and social isolation at age 18 (*r* = 0.24; Fig. 3). Conversely, isolation at age 12 was associated to a lesser extent with depression at age 18 (*r* = 0.15). Correlations with social isolation were generally lower for psychotic experiences and conduct problems, compared to depression, regardless of temporal order.

The IPM (Fig. 2) indicated that common genetic influences substantially contributed to the longitudinal overlap of social isolation with depression symptoms and psychotic experiences. Although non-shared environmental factors influenced the occurrence of each trait individually, they made a small contribution to the overlap between social isolation and mental health problems. Irrespective of the direction of effect, genetic factors contributed to over 80% of the longitudinal association with social isolation for depression symptoms and psychotic experiences, and only 0–30% for conduct problems (Fig. 3). Shared environmental factors contributed substantially to the longitudinal association between social isolation and conduct problems. Across all mental health symptoms, genetic influences contributing to co-occurrence with social isolation were evident at age 12 with limited new genetic influences on co-occurrence emerging at age 18 (A_C2_; Fig. 2). These phenotypic and genetic associations suggest that the underlying genetic predisposition to both social isolation and mental health problems observed at age 18 emerge earlier in development.

There were small differences in the aetiology of the associations based on the direction of the longitudinal association and this differed across disorders (Fig. 3). Genetic influences were more pronounced when childhood conduct problems preceded social isolation in young adulthood (32% increase in A). There was no aetiological distinction based on the direction of the association with social isolation for depression symptoms (2% increase in A) or psychotic experiences (10% increase in A). For conduct problems, 81% of the association was due to the shared environment and 19% due to the non-shared environment.

#### Trait-specific genetic and environmental influences

We noted a few trait-specific observations. First, amongst all traits examined here, heritability was highest for social isolation (39.7-42.5%) (Table 1). Second, shared environmental factors contributed considerably to conduct problems (males: 41.9%-35.2%; females: 25.4-35.2%) but had little influence on all other traits. Third, there were substantial non-shared environmental influences on all traits (55.1-71.4%, which includes measurement error) and these were specific to each trait.

Furthermore, half of the heritability for social isolation at ages 12 and 18 were specific to isolation only (Table 1). Thus, social isolation at each time point has a unique genetic predisposition not shared with mental health symptoms. The proportion of common and specific effects across development differed for depression symptoms and psychotic experiences. Genetic factors underlying depression at age 12 were entirely common across all traits, whereas when children entered adulthood, depression specific genetic effects emerged (37%). Psychotic experiences appeared more heritable at age 12 compared to age 18. These genetic influences were 55% specific to psychosis at age 12 but at age 18 were common across all traits (92%). This pattern hints towards a change in the genetic structure of comorbidity across the lifespan.

**Table 1:** Standardised proportions of common and specific additive genetic, shared environmental, and non-shared environmental influences

	Age 12					Age 18			
	Social Isolation	Depression	Conduct problems (male)	Conduct problems (female)	Psychosis	Social Isolation	Depression	Conduct problems	Psychosis
***h*** ^***2***^	39.69	27.07	1.81	15.89	21.08	42.46	18.94	9.74	9.62
**A**_**C1**_ **%**	43.09	100	100	19.88	45.25	33.12	51.26	0	92.27
**A**_**C2**_ **%**	-	-	-	-	-	9.13	11.83	100	0
**A**_**S**_ **%**	56.91	0	0	80.12	54.76	57.75	36.91	0	7.73
***c*** ^***2***^	3.66	11.23	41.87	25.36	9.49	1.81	9.64	35.18	16.37
**C**_**C1**_ **%**	100	40.37	60.91	100	0	100	7.59	85.10	2.63
**C**_**C2**_ **%**	-	-	-	-	-	0	92.42	14.91	97.37
**C**_**S**_ **%**	0	59.63	39.09	0	100	0	0	0	0
***e*** ^***2***^	56.66	61.70	56.33	58.75	69.44	55.73	71.41	55.08	74.01
**E**_**C1**_ **%**	2.10	7.56	55.09	25.87	3.84	0.78	0.67	8.66	3.38
**E**_**C2**_ **%**	-	-	-	-	-	7.60	26.28	8.42	22.98
**E**_**S**_ **%**	97.90	92.44	44.91	74.14	96.16	91.62	73.05	82.93	73.64

Note. AIC = 9694; h^2^ : heritability estimate; A_C1_%: percentage of h^2^ attributable to common genetic factor 1 (A_C1_ in Fig. 2); A_C2_%: percentage of h^2^ attributable to common genetic factor 2 (A_C2_); A_S_ %: percentage of h^2^ attributable to the specific genetic factors (A_S1−8_); c^2^: standardised shared environment influences; C_C1_%: percentage of c^2^ attributable to common shared environment factor 1 (C_C1_); C_C2_%: percentage of c^2^ attributable to common shared environment factor 2 (C_C2_); C_S_ %: percentage of c^2^ attributable to the specific shared environment factor (C_S1−8_); e^2^: standardised non-shared environment influences; E_C1_%: percentage of e^2^ attributable to common non-shared environment factor 1 (E_C1_); E_C2_%: percentage of e^2^ attributable to common non-shared environment factor 2 (E_C2_); E_S_ %: percentage of e^2^ attributable to the specific non-shared environment factor (E_S1−8_). R functions to compute the independent pathway model provided in Supplement 4. Paths with very small effects were constrained to be zero indicated by ‘-’. Based on preliminary univariate analyses, estimates for conduct problems at age 12 varied by sex.

## Discussion

We investigated common genetic and environmental influences on social isolation and mental health symptoms from childhood to young adulthood using a nationally-representative UK sample. We found that shared genetic risk largely explained the longitudinal overlap between social isolation and mental health symptoms, but the magnitude of this effect varied across disorders and timepoints. Genetically-influenced transdiagnostic risk can account for the longitudinal co-occurrence of social isolation with depression symptoms and psychotic-like experiences, but not for conduct problems. Through a longitudinal genetically sensitive approach, we emphasise moving away from a view that aetiology is static and highlight that social isolation is developmentally intertwined with the experience of poor mental health.

Social experiences are not independent of a person’s characteristics, such as personality or psychopathology. Our hypothesis for a heritable component of social isolation arises from the genetic influences shared with these heritable characteristics. Consistent with previous research on other social behaviours [17, 18], we showed that social isolation was around 50% heritable across childhood. Although social isolation is an environmental experience, the genetic basis likely reflects other heritable characteristics that predispose children to negative interactions with others [19]. Genetic factors contribute to how young people create social connections: individuals self-select and modify their environment or evoke reactions (active and evocative gene-environment correlations, respectively) that can lead to solitary behaviour. This is consistent with other childhood social exposures such as bullying victimisation and other forms of childhood adversity [29]. Children with phenotypic expressions of a genetic liability to mental health symptoms can increase the risk of rejection from people around them.

We found differences in the pattern of genetic and environmental influences on social isolation across development. Genetic factors were prominent early in childhood and contributed to the continuation of social isolation across time. Rather than a specific time period where environmental experiences are most important for social inclusion, unique environmental experiences contributed to developmental changes that were specific to each age. We show evidence for a dynamic aetiology of childhood social isolation, whereby genetic and environmental influences show distinct patterns over time. This pattern is widely reported for childhood internalising, externalising, and withdrawn behaviour [24]. It is possible that the genetic contribution to the stability of social isolation is a reflection of heritable mental health symptoms showing the same developmental pattern.

Our findings suggest that social isolation can be conceptualised as an intertwined component of poor mental health. Rather than a risk factor or an outcome of poor mental health, social isolation could be a marker of functional impairment that occurs alongside mental health symptoms. One comparable example of this type of comorbidity is sleep disturbances. Mental health symptoms are longitudinally and bidirectionally associated with sleep disturbances across childhood and adolescence, and research suggests this is due to underlying risk factors that occur sequentially, in parallel, and interact with each other [30]. Sleep disturbances are distinct from mental health problems, but they typically go hand-in-hand phenotypically and genetically [30]. They are often treated as a peripheral or secondary symptom for mental health disorders. Sleep disturbances can both act as an indicator that a person is having difficulties, as well as subsequently worsen symptoms for people with these disorders. Social isolation and mental health symptoms are interconnected in a similar way to sleep disturbances. Inquiring about social isolation can provide an avenue to identify young people that not only are at risk for mental health symptoms but are already experiencing difficulties that have not yet been recognised.

Our findings support this notion in two ways. First, we found similar patterns of association regardless of the longitudinal direction of effect. For depression symptoms and psychotic experiences, the contribution of genetic and environmental influences was consistent irrespective of whether social isolation was assessed before or after mental health symptoms. This suggests similar mechanisms underlying longitudinal associations, regardless of temporal precedence. Conceptualising social isolation solely as a risk factor could underestimate the complexity of the links to poor mental health. We must note that these findings are based on associations rather than testing causal effects. Qualitative studies have also reported a lack of social connections as integral for people with lived experience of depression, anxiety, and psychosis [31, 32]. A lack of supportive relationships could act as a general indicator or social symptom for mental health problems in young people [11, 13]. Second, genetic factors contributed to over 80% of the association between social isolation and depression symptoms and psychotic experiences. Therefore, previous longitudinal findings could be confounded by common genetic influence. Genetic confounding is well-documented in psychiatric research [33] and has been demonstrated for other childhood environmental exposures [29]. This is one explanation for the mixed findings in the existing literature on the direction of the association between social isolation and mental health symptoms [7], as this association is substantially confounded by shared genetic liabilities across mental health disorders and social behaviours in young people [22]. Genetic overlap suggests intricate interplay between social isolation and mental health symptoms. Our findings emphasise that we cannot assume social isolation is associated with later poor mental health (and vice versa) entirely through an environmental pathway. Instead, social experiences could be considered integral to the phenotypic profile of some psychiatric disorders.

Conduct problems had a distinct aetiological structure which contributed differently to associations with social isolation. Shared environmental factors substantially influenced associations with social isolation, rather than shared genetic influences as for the other mental health symptoms examined here. Children growing up in a violent community or family could be more likely to develop conduct problems and be isolated from their peers [34]. Conduct problems may differentiate from depression and psychotic-like experiences as these atypical behaviours actively shape and maintain social interactions [35]. We show that when conduct problems led to later social isolation, genetic effects contributed 30%, but when this direction was reversed, the genetic effect disappeared. This is consistent with evocative *r*GE: adolescents who are predisposed to developing conduct problems may evoke negative responses from their peer group. Genetic risk could shape young people’s cognitive and affective functioning which inhibits building and maintaining of social relationships [35]. Conduct disorder is highly heterogeneous in its symptomatology and variation in genetic architecture across aggressive and non-aggressive symptoms could explain the lack of genetic overlap [36].

This research has limitations. First, we used different instruments to assess the same constructs over time which could influence model estimates. Particularly for social isolation, variance could be misattributed due to differences in measurement. However, developmental measures must be appropriate for each age and we used clinically relevant measures of mental health symptoms rather than a single child behaviour scale typically used in longitudinal studies. Second, we used only twin-based methods to estimate additive genetic effects which could overestimate genetic effects if the equal environments assumption does not hold (MZ twins treated more similarly compared to DZ twins), when non-additive effects such as gene-gene or gene-environment interactions are being captured. Future research should aim to triangulate DNA-based and twin designs, or move towards a full shift in family-based genomic research which can provide more accurate findings on the developmental aetiology of psychopathology [37]. Third, the six-year age gap between assessments would not have captured dynamic processes between social isolation and mental health. Fourth, our findings on social isolation in a twin sample may not generalise to singletons. As all participants share a sibling of the same age and sex, levels of isolation may be underestimated. However, twins could become isolated from their peers despite, and perhaps because of, closeness with their cotwin. For example, children may withdraw or not seek active socialisation with peers outside the dyad in favour of the company of their cotwin. Fifth, it is unclear how our findings would differ for clinical populations of people with depression, conduct disorder, or psychosis. Therefore, heritability estimates presented here will differ from other samples of different ages, measures, and clinical sampling, but provide insight into developmental processes of these disorders beyond a clinical setting. Sixth, measures of social isolation used in this study were not specifically designed to assess isolation which could limit interpretability. Items were instead selected from the CBCL, an instrument designed to screen for a wide range of emotional and behavioural problems. The selected items broadly capture a reduction in social contact through social withdrawal or peer rejection. Age 18 isolation was operationalised as a lack of social support, as this reflects the extent to which an individual is embedded within a social network. Seventh, experiences of isolation can include feelings of loneliness which could confound the association with mental health problems. However, social isolation takes into consideration an individual’s material social surroundings, rather than focusing on subjective expectations of social connection that can vary from person to person. Identifying structural factors that contribute to a lack of social connectedness shifts away from individual perceptions and identifies areas that are targetable through policy and intervention. Eighth, twin modelling estimates are reliant on a multitude of model specification decisions. Genetic and environmental estimates can vary depending on how many traits are included in the model, how reporter estimates are combined, and which effects are freely estimated. Caution is warranted in interpreting model estimates as true values and replication is needed in other twin cohorts.

Our findings have key implications for clinicians, educational professionals, and future research. First, social isolation can act as an indicator to help identify underlying mental health symptoms in young people. Given the stigma and difficulty to recognise mental health symptoms, assessing social isolation provides an additional way for children to communicate their difficulties. For example, when children report they are consistently by themselves or do not play with any other children at school, this could signal to clinicians other co-occurring difficulties within which social isolation is embedded. The substantial genetic overlap we found does *not* suggest that being isolated is determined by genes, nor that mental health symptoms will always lead to isolation (or vice versa), nor that either cannot be addressed through interventions. Rather, we provide evidence that integrative assessment of social experiences such as isolation may be a helpful add-on to assessments of mental health symptoms, and that any social support interventions for mental health symptoms should be put into practice as early as possible in development. Second, nuanced interdisciplinary research that integrates genetic, social, and developmental approaches into study designs is fundamental to understanding behaviour. Identifying dynamic processes between reciprocal social interactions and psychopathology whilst accounting for genetic confounding is warranted. We demonstrate the value in modelling genetically sensitive longitudinal associations between social isolation and mental health symptoms, that capture both trait overlap and specificity.

## Electronic supplementary material

Below is the link to the electronic supplementary material.

Supplementary Material 1

## Data Availability

The dataset reported in the current article is not publicly available due to lack of informed consent and ethical approval, but is available on request by qualified scientists. Requests require a concept paper describing the purpose of data access, ethical approval at the applicant’s institution, and provision for secure data access. Secure access is offered on the King’s College London campus. For the purposes of open access, the author has applied a Creative Commons Attribution (CC BY) licence to any Accepted Author Manuscript version arising from this submission. All data analysis scripts are available to review on GitHub: http://github.com/knthompson26/isolation_mentalhealth_overlap.
