# Supplementary material for: Social isolation and poor mental health in young people: testing genetic and environmental influences in a longitudinal cohort study
Source: Eur Child Adolesc Psychiatry. 2024 Sep 11;34(4):1445–55. doi: 10.1007/s00787-024-02573-w (PMC12000192; doi:10.1007/s00787-024-02573-w)
Supplement: Supplementary file 1 — Supplementary Material 1 [file 787_2024_2573_MOESM1_ESM.docx]

**Social isolation and poor mental health in young people:**

**Testing genetic and environmental influences in a longitudinal cohort study**

**Supplemental material**

**Katherine N. Thompson^1^, Olakunle Oginni^2, 3^, Jasmin Wertz^4^, Andrea Danese^2,5,6^, Malaika Okundi^2^, Louise Arseneault^2^, and Timothy Matthews^7^**

^1^ Department of Sociology, College of Liberal Arts, Purdue University, West Lafayette, Indiana, United States of America

^2^ Social, Genetic and Developmental Psychiatry Centre, Institute of Psychiatry, Psychology and Neuroscience, King's College London, London, United Kingdom

^3^ Department of Mental Health, Obafemi Awolowo University, Ile-Ife, Nigeria

^4^ Department of Psychology, School of Philosophy, Psychology & Language Sciences, University of Edinburgh, Edinburgh, United Kingdom

^5^ Department of Child & Adolescent Psychiatry, Institute of Psychiatry, Psychology & Neuroscience, King's College London, London, United Kingdom

^6^ National and Specialist CAMHS Trauma, Anxiety, and Depression Clinic, South London and Maudsley NHS Foundation Trust, London, United Kingdom

^7^ School of Human Sciences, Faculty of Education, Health and Human Sciences, University of Greenwich, London, United Kingdom

# Contents

[Contents 2](#_Toc171259741)

[Supplement 1: Supplemental methods 3](#_Toc171259742)

[Part A. E-Risk measures 3](#_Toc171259743)

[Instruments used in the current study 4](#_Toc171259744)

[Part B. Statistical analyses 6](#_Toc171259745)

[The classical twin design 6](#_Toc171259746)

[Cholesky model 6](#_Toc171259747)

[Independent pathway model 7](#_Toc171259748)

[Supplement 2. Results from the Cholesky decomposition for isolation at ages 5, 7, 10, and 12 years 8](#_Toc171259749)

[Part A. Path estimates from the ACE model 8](#_Toc171259750)

[Part B. Path estimates from the AE model 9](#_Toc171259751)

[Part C. ACE and AE model comparison 11](#_Toc171259752)

[Supplement 3. Overlap between social isolation and mental health symptoms 11](#_Toc171259753)

[Part A. Phenotypic correlations between social isolation and mental health symptoms 11](#_Toc171259754)

[Correlations between all mental health problems 11](#_Toc171259755)

[Part B. Univariate ACE models 13](#_Toc171259756)

[No sex differences specified 13](#_Toc171259757)

[Quantitative sex differences specified (heterogeneity model) 13](#_Toc171259758)

[Scalar sex differences (scalar models) 15](#_Toc171259759)

[Comparison across models with different specification for sex differences 16](#_Toc171259760)

[Part C. Independent pathway model 17](#_Toc171259761)

[MZ trait correlations 17](#_Toc171259762)

[DZ trait correlations 18](#_Toc171259763)

[Means and standard deviations for all variables 18](#_Toc171259764)

[Unconstrained independent pathway model 19](#_Toc171259765)

[Female only contributions to the independent pathway model 20](#_Toc171259766)

[Part D. Post hoc sensitivity analysis for the association between social isolation and conduct problems 21](#_Toc171259767)

[Supplement 4: R functions to replicate independent pathway model 27](#_Toc171259768)

[References 28](#_Toc171259769)

#

# Supplement 1: Supplemental methods

## Part A. E-Risk measures

The E-Risk sample represents socioeconomic conditions in the UK, as reflected in the families’ distribution on neighbourhood-level socioeconomic indices (**Figure S1**).


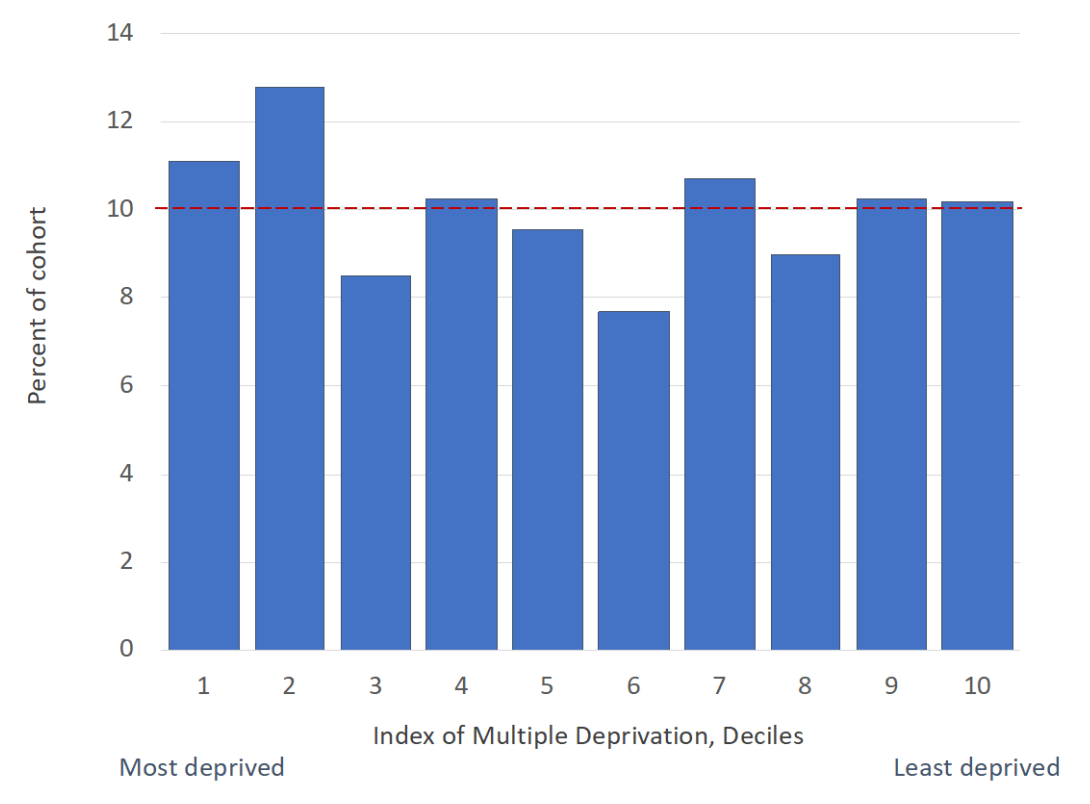


**Figure S1.** Population representativeness of the E-Risk sample from (Reuben et al., 2020). The histogram shows E-Risk families’ addresses are a near-perfect match to the deciles of the UK’s 2015 Lower-layer Super Output Area (LSOA) Index of Multiple Deprivation (IMD) which averages 1,500 residents; approximately 10% of the E-Risk cohort (dotted red line) fills each of the IMD’s 10% bands, indicating that the E-Risk cohort accurately represents the distribution of deprivation in the UK.

#### Instruments used in the current study

| **Table S1.** Description of the measures of social isolation and mental health problems | | | | | |
| --- | --- | --- | --- | --- | --- |
|  | **Age** | **Instrument** | **Reporter** | **Description** | **Reference** |
| **Social isolation** | 5,  7, 10, &  12 | Child Behaviour Checklist (CBCL) and Teacher Report Form (TRF) | Combined mother and teacher report | We conceptualised social isolation in childhood as a combination of limited interactions with other children, social rejection, and withdrawal (Caspi et al., 2006; Matthews et al., 2015; Thompson et al., 2022, 2023), using six items including whether the child ‘would rather be alone than with others’, ‘was not liked by other children [pupils]’, ‘does not get along with other children [pupils]’, ‘feels or complains that no-one loves him/her’, ‘is withdrawn, does not get involved with others’ and ‘complains of loneliness’. Responses were scored “not true” (0), “somewhat true” (1), and “often true” (2). Items were summed to create mother and teacher scales at each age (*r* at each age = 0.26-0.31). These were then averaged across mothers and teachers to give a combined scale which integrates observations from different settings (α=0.68-0.78). This conceptualisation of social isolation reflects common experiences within the general population rather than extreme forms of neglect as typically measured in animal studies. Mothers completed the questionnaire in a face-to-face interview at each age and teachers responded to the same items by post. From age 5 to 12, 2079 (93.14%) children had complete social isolation data at all time points. Missingness information for all measures is provided in **Table S2**. | (Achenbach, 1991; Achenbach & Edelbrock, 1991; Caspi et al., 2006; Matthews et al., 2015; Thompson et al., 2022, 2023) |
|  | 18 | Multidimensional Scale of Perceived Social Support (12 items; MSPSS) | Self-report | MSPSS reflects an individuals’ access to supportive relationships with family and friends. We used low social support as a proxy for adult social isolation, as indicators such as marital status or living alone were not applicable to the majority of 18-year olds in E-Risk. 12 self-report items which consist of statements such as ‘‘there is a special person who is around when I am in need’’ and ‘‘I can count on my friends when things go wrong’’. Participants rated these statements as ‘‘not true’’ (0), ‘‘somewhat true’’ (1) or ‘‘very true’’ (2). We reversed the scoring and summed the responses to produce a scale with high scores reflecting greater social isolation (α=0.88). | (Matthews et al., 2016; Zimet et al., 1988) |
| **Depression symptoms** | 12 | Children’s Depression Inventory (CDI) | Self-report | Each child was asked to choose one statement out of three that best described their feelings over the past two weeks. Each symptom statement increased with severity and was coded 0, 1, or 2 by the researcher. Statements covered 27 items such as “feel sad”, “hate myself”, “feel like crying”, “bad things happening”, and “bad things my fault”. The 10-item short-form version was first administered as screening questions (CDI-S). For children who scored more than 5 (indicating possible depression), the remaining 17 items were administered. | (Kovacs, 1985) |
|  | 18 | Structured interview based on the Diagnostic Interview Schedule | Self-report | Participants were asked four questions on persistent low mood, anhedonia, irritability, and prescribed depression medication in the last year, and a further 24 questions which mapped directly onto the remaining symptoms of a major depressive episode specified in the Diagnostic and Statistical Manual of Mental Disorders, Fourth Edition (DSM-IV). We summed endorsement of these items to provide a score for depression at age 18. | (American Psychiatric Association, 1994; Robins et al., 1995) |
| **Conduct problems** | 12  &  18 | Computerised questionnaire of antisocial behaviours based on DSM-IV criteria for conduct disorder | Self-report | At both ages, items included the use of weapons (e.g., “have you used a weapon on someone like a knife, piece of wood, or baseball bat?”), truancy (e.g., “do you sometimes skip school when you shouldn’t?”), and stealing (e.g., “have you stolen something while nobody was looking?”). Children responded with “yes” or “no” and were able to refuse to answer. At age 18, an additional 20 items were asked around age-related antisocial behaviours such as “have you stolen a car or motorcycle?” or “have you sold hard drugs?”. In some cases, more than one item was used to map onto the same DSM-IV symptom. We summed the number of symptoms present at each age to provide a score for conduct problems. | (American Psychiatric Association, 1994) |
| **Psychotic-like experiences** | 12 & 18 | Structured interview on hallucinations and delusions | Self-report | Seven items such as “have you ever believed that you were sent special messages through TV or radio?”, “have other people ever read your thoughts?”, “have you ever seen something or someone that other people could not see?”, “have you ever thought you were being watched, followed, or spied on?”, and “do you hear voices that others cannot?”. Interviewers coded each experience “not a symptom”/“not present” (0), “probable symptom”/“probably present” (1), and “definite symptom”/“definitely present” (2). Items were summed to create a psychotic-like experiences scale at each age. At age 12, items and interviewer notes were verified by a psychiatrist expert in schizophrenia, psychologist expert in interviewing children, and a child and adolescent psychiatrist. At age 18, an additional set of six items were included to assess other unusual experiences, such as “I believe I have special abilities or powers beyond my natural talents”. When a child endorsed any symptom, the interviewer probed using standard prompts designed to discriminate between experiences that were plausibly real (e.g., “I was followed by a man after school”) and potential symptoms (e.g., “I was followed by an angel who guards my spirit”) and wrote down the child’s narrative description of the experience. Experiences limited to the twin relationship (e.g., “my twin and I often know what each other are thinking”) were coded as “not a symptom”. | (American Psychiatric Association, 1994; Newbury et al., 2023; Polanczyk et al., 2010) |

##

| **Table S2**. Missing data for all phenotypic variables at age 12 and 18 | | | |
| --- | --- | --- | --- |
|  | **Complete data (n)** | **Missing (n)** | **Total n answered**  **(Grand N = 2232)** |
| Social isolation age 12 | 2145* | 87 | 2143 |
| Social isolation age 18 | 2061 | 171 | 2076 |
| Depression symptoms age 12 | 2130 | 102 | 2143 |
| Depression symptoms age 18 | 2063 | 169 | 2076 |
| Conduct problems age 12 | 2120 | 112 | 2143 |
| Conduct problems age 18 | 2053 | 179 | 2076 |
| Psychotic experiences age 12 | 2127 | 105 | 2143 |
| Psychotic experiences age 18 | 2063 | 169 | 2076 |
| *Above the total responses as this is combined with teacher items that were returned by post | | | |

## Part B. Statistical analyses

#### The classical twin design

When correlations within MZ twin pairs for a phenotype are higher than correlations within DZ pairs, this suggests there are genetic influences on this trait as both MZ and DZ twin pairs experience the same shared environment. To estimate distinct ACE components, we assume that genetic correlations between MZ twins are 1, DZ twins are 0.5, and shared environmental correlations are 1 for both MZ and DZ twins. Any differences between MZ twins inform the estimation of E, which includes phenotype measurement error.

#### Cholesky model

Cholesky models assume a logical order in which the variables are entered, such as temporality. As such, it allows for ACE influences on a variable measured at an initial time point to also influence variable(s) measured at subsequent time points, as well as estimating new ACE effects later in time. Thus, each variable can be influenced by ACE factors underlying the variables that precede it, but not vice versa (Loehlin, 1996).

#### Independent pathway model

The independent pathway model specifies a common set of additive genetic (A), shared environmental (C) and non-shared environmental (E) influences which differentially influence all the variables of interest. In addition to these, each variable is further influenced by a set of ACE influences that are unique to it (i.e., not shared with other variables in the model). For the present analyses we specified two sets of common ACE influences: one was shared by all the variables at ages 12 and 18 while the second set was shared only by variables at age 18.

*Sex differences*

We tested for quantitative and scalar sex differences for all traits. Quantitative sex differences indicate that different proportions of the same A, C, and E contribute to a trait for males and females. Scalar sex differences indicate that sex differences reflect differences in phenotypic variance rather than true quantitative sex differences. These sex differences were preliminarily explored in univariate models which estimated ACE influences on each variable separately. Quantitative sex differences were demonstrated only for conduct problems at age 12 while scalar differences were demonstrated for all the other variables, and these were incorporated into the multivariate models.

# Supplement 2. Results from the Cholesky decomposition for isolation at ages 5, 7, 10, and 12 years

## Part A. Path estimates from the ACE model

| **Table S3.** All standardised path estimates for the Cholesky decomposition ACE model. | | | | |
| --- | --- | --- | --- | --- |
| **ACE** | **Age** | **Confidence interval**  **Lower bound** | **Estimate** | **Confidence interval**  **Upper bound** |
| h2 | 5 | 0.500 | 0.561 | 0.615 |
| h2 | 7 | 0.394 | 0.493 | 0.553 |
| h2 | 10 | 0.371 | 0.487 | 0.547 |
| h2 | 12 | 0.350 | 0.445 | 0.505 |
| c2 | 5 | 0.000 | 0.000 | 0.024 |
| c2 | 7 | 0.000 | 0.001 | 0.069 |
| c2 | 10 | 0.000 | 0.002 | 0.089 |
| c2 | 12 | 0.000 | 0.001 | 0.068 |
| e2 | 5 | 0.385 | 0.439 | 0.500 |
| e2 | 7 | 0.447 | 0.506 | 0.572 |
| e2 | 10 | 0.453 | 0.511 | 0.574 |
| e2 | 12 | 0.494 | 0.554 | 0.619 |
| sta2 | 5 to 7 | 0.135 | 0.192 | 0.251 |
| stc2 | 5 to 7 | 0.000 | 0.001 | 0.069 |
| ste2 | 5 to 7 | 0.039 | 0.065 | 0.097 |
| sta2 | 5 to 10 | 0.098 | 0.148 | 0.208 |
| stc2 | 5 to 10 | 0.000 | 0.002 | 0.089 |
| ste2 | 5 to 10 | 0.004 | 0.015 | 0.032 |
| sta2 | 5 to 12 | 0.087 | 0.134 | 0.188 |
| stc2 | 5 to 12 | 0.000 | 0.001 | 0.068 |
| ste2 | 5 to 12 | 0.000 | 0.006 | 0.019 |
| sta2 | 7 to 10 | 0.079 | 0.137 | 0.254 |
| stc2 | 7 to 10 | 0.000 | 0.000 | 0.087 |
| ste2 | 7 to 10 | 0.009 | 0.023 | 0.043 |
| sta2 | 7 to 12 | 0.021 | 0.064 | 0.095 |
| stc2 | 7 to 12 | 0.000 | 0.000 | 0.065 |
| ste2 | 7 to 12 | 0.008 | 0.022 | 0.042 |
| sta2 | 10 to 12 | 0.058 | 0.130 | 0.293 |
| stc2 | 10 to 12 | 0.000 | 0.000 | 0.058 |
| ste2 | 10 to 12 | 0.031 | 0.053 | 0.080 |
| sta2 | 5 | 0.500 | 0.561 | 0.615 |
| stc2 | 5 | 0.000 | 0.000 | 0.024 |
| ste2 | 5 | 0.385 | 0.439 | 0.500 |
| sta2 | 7 | 0.216 | 0.301 | 0.359 |
| stc2 | 7 | 0.000 | 0.000 | 0.054 |
| ste2 | 7 | 0.390 | 0.441 | 0.498 |
| sta2 | 10 | 0.051 | 0.202 | 0.264 |
| stc2 | 10 | 0.000 | 0.000 | 0.087 |
| ste2 | 10 | 0.421 | 0.473 | 0.530 |
| sta2 | 12 | 0.000 | 0.118 | 0.173 |
| stc2 | 12 | 0.000 | 0.000 | 0.057 |
| ste2 | 12 | 0.424 | 0.474 | 0.527 |
| *Note*. ACE: additive genetic, shared environment, and non-shared environment; h2: heritability estimate; c2: shared environment estimate; e2: non-shared environment estimate; sta2: standardised additive genetic path estimate; stc2: standardised shared environment path estimate; ste2: standardised non-shared environment path estimate. | | | | |

## Part B. Path estimates from the AE model

| **Table S4.** All standardised path estimates for the Cholesky decomposition AE model. | | | | |
| --- | --- | --- | --- | --- |
| **ACE** | **Age** | **Confidence interval**  **Lower bound** | **Estimate** | **Confidence interval**  **Upper bound** |
| h2 | 5 | 0.500 | 0.561 | 0.615 |
| h2 | 7 | 0.429 | 0.494 | 0.553 |
| h2 | 10 | 0.427 | 0.490 | 0.547 |
| h2 | 12 | 0.381 | 0.446 | 0.505 |
| c2 | 5 | *NA* | 0.000 | *NA* |
| c2 | 7 | *NA* | 0.000 | *NA* |
| c2 | 10 | *NA* | 0.000 | *NA* |
| c2 | 12 | *NA* | 0.000 | *NA* |
| e2 | 5 | 0.385 | 0.439 | 0.500 |
| e2 | 7 | 0.447 | 0.506 | 0.571 |
| e2 | 10 | 0.453 | 0.510 | 0.573 |
| e2 | 12 | 0.495 | 0.554 | 0.619 |
| sta2 | 5 to 7 | 0.138 | 0.192 | 0.251 |
| stc2 | 5 to 7 | *NA* | 0.000 | *NA* |
| ste2 | 5 to 7 | 0.039 | 0.065 | 0.097 |
| sta2 | 5 to 10 | 0.100 | 0.148 | 0.203 |
| stc2 | 5 to 10 | *NA* | 0.000 | *NA* |
| ste2 | 5 to 10 | 0.004 | 0.015 | 0.032 |
| sta2 | 5 to 12 | 0.088 | 0.134 | 0.187 |
| stc2 | 5 to 12 | *NA* | 0.000 | *NA* |
| ste2 | 5 to 12 | 0.000 | 0.006 | 0.019 |
| sta2 | 7 to 10 | 0.083 | 0.135 | 0.196 |
| stc2 | 7 to 10 | *NA* | 0.000 | *NA* |
| ste2 | 7 to 10 | 0.009 | 0.023 | 0.043 |
| sta2 | 7 to 12 | 0.028 | 0.064 | 0.112 |
| stc2 | 7 to 12 | *NA* | 0.000 | *NA* |
| ste2 | 7 to 12 | 0.008 | 0.022 | 0.042 |
| sta2 | 10 to 12 | 0.071 | 0.126 | 0.191 |
| stc2 | 10 to 12 | *NA* | 0.000 | *NA* |
| ste2 | 10 to 12 | 0.031 | 0.053 | 0.081 |
| sta2 | 5 | 0.500 | 0.561 | 0.615 |
| stc2 | 5 | *NA* | 0.000 | *NA* |
| ste2 | 5 | 0.385 | 0.439 | 0.500 |
| sta2 | 7 | 0.242 | 0.302 | 0.359 |
| stc2 | 7 | *NA* | 0.000 | *NA* |
| ste2 | 7 | 0.390 | 0.441 | 0.498 |
| sta2 | 10 | 0.147 | 0.206 | 0.263 |
| stc2 | 10 | *NA* | 0.000 | *NA* |
| ste2 | 10 | 0.421 | 0.473 | 0.529 |
| sta2 | 12 | 0.069 | 0.122 | 0.173 |
| stc2 | 12 | *NA* | 0.000 | *NA* |
| ste2 | 12 | 0.424 | 0.474 | 0.527 |
| *Note*. ACE: additive genetic, shared environment, and non-shared environment; h2: heritability estimate; c2: shared environment estimate; e2: non-shared environment estimate; sta2: standardised additive genetic path estimate; stc2: standardised shared environment path estimate; ste2: standardised non-shared environment path estimate. | | | | |

## Part C. ACE and AE model comparison

| **Table S5.** Comparison of fit across ACE and AE Choleksy decomposition models | | | | | | | | |
| --- | --- | --- | --- | --- | --- | --- | --- | --- |
| **Base model** | **Comparison model** | **ep** | **minus2LL** | **df** | **AIC** | **diffLL** | **diffdf** | ***p*** |
| ACE |  | 34 | 22000.94 | 8659 | 22068.94 | *-* | *-* | *-* |
| ACE | AE | 24 | 22000.95 | 8669 | 22048.95 | 0.006200344 | 10 | 1 |

# Supplement 3. Overlap between social isolation and mental health symptoms

## Part A. Phenotypic correlations between social isolation and mental health symptoms

###

### Correlations between all mental health problems

To be included in the independent pathway model, we applied a cut off of 0.1 for the correlations between social isolation and all mental health symptoms, to enable us to partition this into A, C, and E parameters. This is because smaller correlations will be difficult to resolve into ACE components. To capture a broad range of mental health problems, we initially intended to include anxiety items in our analyses. However, phenotypic correlations anxiety at age 12 and 18 unexpectedly showed low correlations with social isolation and with the other variables (except depressive symptoms; **Figure S2**). To double check that anxiety was not associated with social isolation, we investigated the factor structure of all variables by specifying the phenotypic equivalent of the IPM and inspecting the factor loadings (**Table S6**). Of these, the standardised factor loading was lowest for Anxiety at age 18 (0.196). This suggests that anxiety is not contributing to the common variance between social isolation and mental health problems in our sample. Due to the low correlations with the other variables and this small factor loading, we decided to exclude Anxiety at both timepoints from the model. Thus, we removed anxiety from our list of mental health symptoms before conducting the IPM ACE analyses and anxiety is not reported on in the main text.


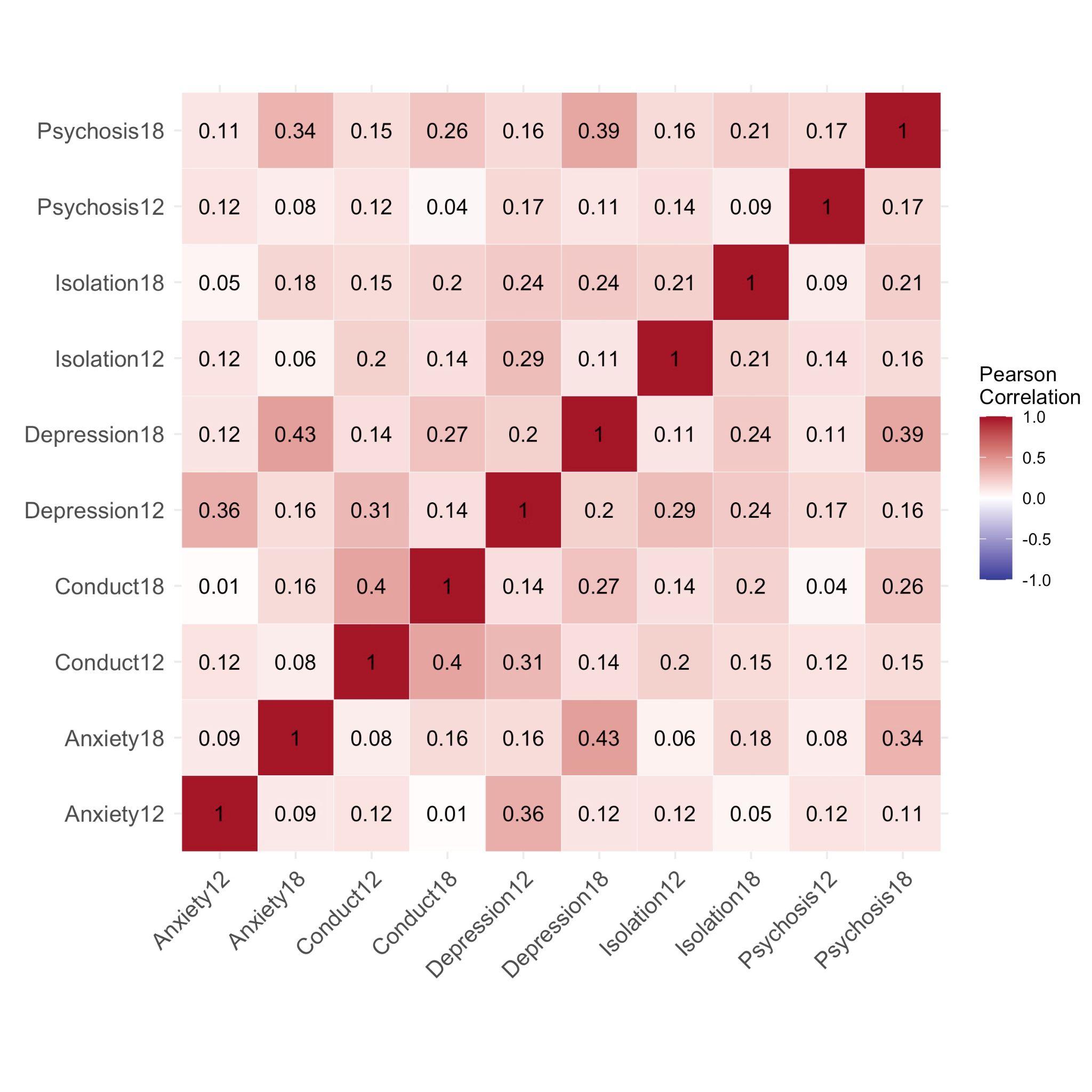


**Figure S2**. Correlation heat map for all variables: social isolation, depression symptoms, anxiety symptoms, conduct problems, and psychotic-like experiences at ages 12 and 18.

| **Table S6.** Factor loadings for the phenotypic IPM including anxiety | | |
| --- | --- | --- |
| **Factor** | **Variable** | **Standardised factor loading** |
| Common all | Isolation12 | 0.439 |
| Common all | Anxiety12 | 0.353 |
| Common all | Depression12 | 0.618 |
| Common all | Conduct12 | 0.532 |
| Common all | Psychosis12 | 0.283 |
| Common all | Isolation18 | 0.363 |
| Common all | Anxiety18 | 0.196 |
| Common all | Depression18 | 0.296 |
| Common all | Conduct18 | 0.389 |
| Common all | Psychosis18 | 0.301 |
| Common age 18 | Isolation18 | 0.418 |
| Common age 18 | Anxiety18 | 0.592 |
| Common age 18 | Depression18 | 0.719 |
| Common age 18 | Conduct18 | 0.448 |
| Common age 18 | Psychosis18 | 0.580 |

## Part B. Univariate ACE models

### No sex differences specified

| **Table S7.** Heritability, shared environment, and non-shared environment estimates for all variables as produced by univariate analyses. | | | | | |
| --- | --- | --- | --- | --- | --- |
| **Variable** | **Age** | **ACE** | **Lower bound** | **Estimate** | **Upper bound** |
| Social isolation | 12 | h2 | 0.334 | 0.419 | 0.481 |
| Social isolation | 12 | c2 | 0.000 | 0.000 | 0.056 |
| Social isolation | 12 | e2 | 0.519 | 0.581 | 0.649 |
| Depression | 12 | h2 | 0.001 | 0.211 | 0.412 |
| Depression | 12 | c2 | 0.000 | 0.146 | 0.318 |
| Depression | 12 | e2 | 0.577 | 0.643 | 0.715 |
| Conduct disorder | 12 | h2 | 0.005 | 0.197 | 0.397 |
| Conduct disorder | 12 | c2 | 0.073 | 0.249 | 0.410 |
| Conduct disorder | 12 | e2 | 0.496 | 0.554 | 0.617 |
| Psychosis | 12 | h2 | 0.000 | 0.185 | 0.383 |
| Psychosis | 12 | c2 | 0.000 | 0.129 | 0.292 |
| Psychosis | 12 | e2 | 0.610 | 0.686 | 0.769 |
| Social isolation | 18 | h2 | 0.346 | 0.450 | 0.510 |
| Social isolation | 18 | c2 | 0.000 | 0.000 | 0.000 |
| Social isolation | 18 | e2 | 0.490 | 0.550 | 0.615 |
| Depression | 18 | h2 | 0.216 | 0.315 | 0.383 |
| Depression | 18 | c2 | 0.000 | 0.000 | 0.072 |
| Depression | 18 | e2 | 0.617 | 0.685 | 0.756 |
| Conduct disorder | 18 | h2 | 0.004 | 0.195 | 0.395 |
| Conduct disorder | 18 | c2 | 0.093 | 0.270 | 0.432 |
| Conduct disorder | 18 | e2 | 0.477 | 0.535 | 0.598 |
| Psychosis | 18 | h2 | 0.000 | 0.237 | 0.347 |
| Psychosis | 18 | c2 | 0.000 | 0.042 | 0.252 |
| Psychosis | 18 | e2 | 0.653 | 0.721 | 0.794 |

### Quantitative sex differences specified (heterogeneity model)

| **Table S8.** Univariate heterogeneity ACE models for all variables | | | | | |
| --- | --- | --- | --- | --- | --- |
| **Variable** | **Age** | **ACE** | **Lower bound** | **Estimate** | **Upper bound** |
| Social isolation | 12 | h2m | 0.277 | 0.417 | 0.500 |
| Social isolation | 12 | c2m | 0.000 | 0.000 | 0.107 |
| Social isolation | 12 | e2m | 0.500 | 0.583 | 0.674 |
| Social isolation | 12 | h2f | 0.278 | 0.429 | 0.522 |
| Social isolation | 12 | c2f | 0.000 | 0.000 | 0.096 |
| Social isolation | 12 | e2f | 0.478 | 0.571 | 0.677 |
| Depression | 12 | h2m | 0.000 | 0.208 | 0.445 |
| Depression | 12 | c2m | 0.000 | 0.147 | 0.363 |
| Depression | 12 | e2m | 0.551 | 0.646 | 0.751 |
| Depression | 12 | h2f | 0.000 | 0.223 | 0.449 |
| Depression | 12 | c2f | 0.000 | 0.139 | 0.361 |
| Depression | 12 | e2f | 0.548 | 0.638 | 0.738 |
| Conduct disorder | 12 | h2m | 0.000 | 0.210 | 0.488 |
| Conduct disorder | 12 | c2m | 0.012 | 0.256 | 0.463 |
| Conduct disorder | 12 | e2m | 0.452 | 0.534 | 0.629 |
| Conduct disorder | 12 | h2f | 0.000 | 0.149 | 0.437 |
| Conduct disorder | 12 | c2f | 0.000 | 0.225 | 0.406 |
| Conduct disorder | 12 | e2f | 0.540 | 0.626 | 0.720 |
| Psychosis | 12 | h2m | 0.000 | 0.225 | 0.432 |
| Psychosis | 12 | c2m | 0.000 | 0.106 | 0.325 |
| Psychosis | 12 | e2m | 0.568 | 0.669 | 0.783 |
| Psychosis | 12 | h2f | 0.000 | 0.000 | 0.220 |
| Psychosis | 12 | c2f | 0.067 | 0.243 | 0.322 |
| Psychosis | 12 | e2f | 0.664 | 0.757 | 0.840 |
| Social isolation | 18 | h2m | 0.304 | 0.451 | 0.538 |
| Social isolation | 18 | c2m | 0.000 | 0.000 | 0.109 |
| Social isolation | 18 | e2m | 0.462 | 0.549 | 0.647 |
| Social isolation | 18 | h2f | 0.215 | 0.447 | 0.528 |
| Social isolation | 18 | c2f | 0.000 | 0.000 | 0.195 |
| Social isolation | 18 | e2f | 0.472 | 0.553 | 0.643 |
| Depression | 18 | h2m | 0.096 | 0.271 | 0.370 |
| Depression | 18 | c2m | 0.000 | 0.000 | 0.132 |
| Depression | 18 | e2m | 0.630 | 0.729 | 0.835 |
| Depression | 18 | h2f | 0.183 | 0.345 | 0.435 |
| Depression | 18 | c2f | 0.000 | 0.000 | 0.124 |
| Depression | 18 | e2f | 0.565 | 0.655 | 0.752 |
| Conduct disorder | 18 | h2m | 0.004 | 0.279 | 0.553 |
| Conduct disorder | 18 | c2m | 0.000 | 0.216 | 0.449 |
| Conduct disorder | 18 | e2m | 0.427 | 0.505 | 0.594 |
| Conduct disorder | 18 | h2f | 0.000 | 0.096 | 0.374 |
| Conduct disorder | 18 | c2f | 0.093 | 0.336 | 0.477 |
| Conduct disorder | 18 | e2f | 0.485 | 0.567 | 0.655 |
| Psychosis | 18 | h2m | 0.000 | 0.267 | 0.363 |
| Psychosis | 18 | c2m | 0.000 | 0.000 | 0.000 |
| Psychosis | 18 | e2m | 0.637 | 0.733 | 0.838 |
| Psychosis | 18 | h2f | 0.000 | 0.125 | 0.373 |
| Psychosis | 18 | c2f | 0.000 | 0.160 | 0.342 |
| Psychosis | 18 | e2f | 0.624 | 0.715 | 0.811 |

### Scalar sex differences (scalar models)

| **Table S9.** Univariate ACE models with scalars applied to account for differences in total variance across males and females. | | | | | |
| --- | --- | --- | --- | --- | --- |
| **Variable** | **Age** | **ACE** | **Lower bound** | **Estimate** | **Upper bound** |
| Social isolation | 12 | h2 | 0.337 | 0.422 | 0.485 |
| Social isolation | 12 | c2 | 0.000 | 0.000 | 0.056 |
| Social isolation | 12 | e2 | 0.515 | 0.578 | 0.646 |
| Depression | 12 | h2 | 0.005 | 0.216 | 0.414 |
| Depression | 12 | c2 | 0.000 | 0.143 | 0.315 |
| Depression | 12 | e2 | 0.575 | 0.641 | 0.713 |
| Conduct disorder | 12 | h2 | 0.000 | 0.172 | 0.375 |
| Conduct disorder | 12 | c2 | 0.069 | 0.245 | 0.406 |
| Conduct disorder | 12 | e2 | 0.522 | 0.583 | 0.649 |
| Psychosis | 12 | h2 | 0.000 | 0.058 | 0.279 |
| Psychosis | 12 | c2 | 0.041 | 0.215 | 0.312 |
| Psychosis | 12 | e2 | 0.647 | 0.726 | 0.798 |
| Social isolation | 18 | h2 | 0.342 | 0.449 | 0.509 |
| Social isolation | 18 | c2 | 0.000 | 0.000 | 0.000 |
| Social isolation | 18 | e2 | 0.491 | 0.551 | 0.616 |
| Depression | 18 | h2 | 0.211 | 0.310 | 0.378 |
| Depression | 18 | c2 | 0.000 | 0.000 | 0.071 |
| Depression | 18 | e2 | 0.622 | 0.690 | 0.761 |
| Conduct disorder | 18 | h2 | 0.000 | 0.184 | 0.384 |
| Conduct disorder | 18 | c2 | 0.102 | 0.279 | 0.440 |
| Conduct disorder | 18 | e2 | 0.479 | 0.537 | 0.600 |
| Psychosis | 18 | h2 | 0.000 | 0.230 | 0.347 |
| Psychosis | 18 | c2 | 0.000 | 0.049 | 0.260 |
| Psychosis | 18 | e2 | 0.653 | 0.721 | 0.793 |

### Comparison across models with different specification for sex differences

From the AIC estimates for the homogeneity (no sex differences), heterogeneity (separate paths), and scalar models (scaled variances), the scalar model was the best fit to the data for all variables apart from depression at age 12 and conduct problems at age 12 (**Table S10**). For depression at age 12, the homogeneity model was best fit. For conduct problems at age 12, the heterogeneity model was best fit. Therefore, when specifying the independent pathway model, separate paths for males and females were computed for conduct problems to account for quantitative sex differences. Apart from depression symptoms at age 12, all remaining variances were scaled to account for proportional sex differences.

| **Table S10.** AIC values for all univariate models (homogeneity, heterogeneity, and scalar). | | |
| --- | --- | --- |
| **Variable** | **Model** | **AIC** |
| Isolation12 | Het | 5957.900 |
| Isolation12 | Homo | 5962.149 |
| Isolation12 | Scalar | 5953.929 |
| Depression12 | Het | 5612.801 |
| Depression12 | Homo | 5605.325 |
| Depression12 | Scalar | 5608.814 |
| Conduct12 | Het | 5721.944 |
| Conduct12 | Homo | 5733.520 |
| Conduct12 | Scalar | 5723.206 |
| Psychosis12 | Het | 5911.270 |
| Psychosis12 | Homo | 5967.309 |
| Psychosis12 | Scalar | 5909.324 |
| Isolation18 | Het | 5697.203 |
| Isolation18 | Homo | 5700.345 |
| Isolation18 | Scalar | 5693.206 |
| Depression18 | Het | 4581.946 |
| Depression18 | Homo | 4601.350 |
| Depression18 | Scalar | 4579.041 |
| Conduct18 | Het | 5513.306 |
| Conduct18 | Homo | 5512.337 |
| Conduct18 | Scalar | 5510.515 |
| Psychosis18 | Het | 5790.523 |
| Psychosis18 | Homo | 5798.904 |
| Psychosis18 | Scalar | 5787.438 |
| **Note.** Highlighted in green are the best fitting models according to the AIC. | | |

## Part C. Independent pathway model

###

### MZ trait correlations

| **Table S11**. MZ trait correlations | | | | | | | | | | | | | | | | | |
| --- | --- | --- | --- | --- | --- | --- | --- | --- | --- | --- | --- | --- | --- | --- | --- | --- | --- |
|  |  | Elder twin (Twin 1) | | | | | | | | Younger twin (Twin 2) | | | | | | | |
|  |  | si12 | d12 | c12 | p12 | si18 | d18 | c18 | p18 | si12 | d12 | c12 | p12 | si18 | d18 | c18 | p18 |
| Elder twin | si12 | 1.000 | 0.239 | 0.195 | 0.124 | 0.201 | 0.096 | 0.207 | 0.173 | 0.420 | 0.172 | 0.143 | 0.010 | 0.205 | 0.109 | 0.163 | 0.101 |
|  | d12 | 0.239 | 1.000 | 0.212 | 0.163 | 0.238 | 0.160 | 0.129 | 0.176 | 0.185 | 0.265 | 0.161 | 0.135 | 0.135 | 0.171 | 0.170 | 0.135 |
|  | c12 | 0.195 | 0.212 | 1.000 | 0.117 | 0.152 | 0.082 | 0.477 | 0.130 | 0.170 | 0.163 | 0.525 | 0.108 | 0.155 | 0.015 | 0.370 | 0.080 |
|  | p12 | 0.124 | 0.163 | 0.117 | 1.000 | 0.097 | 0.067 | 0.046 | 0.104 | 0.101 | 0.106 | 0.075 | 0.255 | 0.093 | 0.070 | 0.059 | 0.056 |
|  | si18 | 0.201 | 0.238 | 0.152 | 0.097 | 1.000 | 0.244 | 0.219 | 0.157 | 0.122 | 0.204 | 0.130 | 0.065 | 0.455 | 0.099 | 0.205 | 0.106 |
|  | d18 | 0.096 | 0.160 | 0.082 | 0.067 | 0.244 | 1.000 | 0.209 | 0.430 | 0.104 | 0.195 | 0.048 | 0.104 | 0.127 | 0.336 | 0.143 | 0.204 |
|  | c18 | 0.207 | 0.129 | 0.477 | 0.046 | 0.219 | 0.209 | 1.000 | 0.259 | 0.195 | 0.111 | 0.374 | 0.020 | 0.193 | 0.085 | 0.536 | 0.084 |
|  | p18 | 0.173 | 0.176 | 0.130 | 0.104 | 0.157 | 0.430 | 0.259 | 1.000 | 0.077 | 0.171 | 0.116 | 0.092 | 0.105 | 0.284 | 0.176 | 0.280 |
| Younger twin | si12 | 0.420 | 0.185 | 0.170 | 0.101 | 0.122 | 0.104 | 0.195 | 0.077 | 1.000 | 0.252 | 0.201 | 0.042 | 0.207 | 0.075 | 0.182 | 0.071 |
|  | d12 | 0.172 | 0.265 | 0.163 | 0.106 | 0.204 | 0.195 | 0.111 | 0.171 | 0.252 | 1.000 | 0.254 | 0.338 | 0.196 | 0.227 | 0.101 | 0.183 |
|  | c12 | 0.143 | 0.161 | 0.525 | 0.075 | 0.130 | 0.048 | 0.374 | 0.116 | 0.201 | 0.254 | 1.000 | 0.230 | 0.222 | 0.059 | 0.463 | 0.147 |
|  | p12 | 0.010 | 0.135 | 0.108 | 0.255 | 0.065 | 0.104 | 0.020 | 0.092 | 0.042 | 0.338 | 0.230 | 1.000 | 0.070 | 0.160 | 0.065 | 0.200 |
|  | si18 | 0.205 | 0.135 | 0.155 | 0.093 | 0.455 | 0.127 | 0.193 | 0.105 | 0.207 | 0.196 | 0.222 | 0.070 | 1.000 | 0.140 | 0.272 | 0.221 |
|  | d18 | 0.109 | 0.171 | 0.015 | 0.070 | 0.099 | 0.336 | 0.085 | 0.284 | 0.075 | 0.227 | 0.059 | 0.160 | 0.140 | 1.000 | 0.189 | 0.410 |
|  | c18 | 0.163 | 0.170 | 0.370 | 0.059 | 0.205 | 0.143 | 0.536 | 0.176 | 0.182 | 0.101 | 0.463 | 0.065 | 0.272 | 0.189 | 1.000 | 0.341 |
|  | p18 | 0.101 | 0.135 | 0.080 | 0.056 | 0.106 | 0.204 | 0.084 | 0.280 | 0.071 | 0.183 | 0.147 | 0.200 | 0.221 | 0.410 | 0.341 | 1.000 |

###

### DZ trait correlations

| **Table S12**. DZ trait correlations | | | | | | | | | | | | | | | | | |
| --- | --- | --- | --- | --- | --- | --- | --- | --- | --- | --- | --- | --- | --- | --- | --- | --- | --- |
|  |  | Elder twin (Twin 1) | | | | | | | | Younger twin (Twin 2) | | | | | | | |
|  |  | si12 | d12 | c12 | p12 | si18 | d18 | c18 | p18 | si12 | d12 | c12 | p12 | si18 | d18 | c18 | p18 |
| Elder twin | si12 | 1.000 | 0.383 | 0.308 | 0.271 | 0.288 | 0.140 | 0.149 | 0.290 | 0.147 | 0.149 | 0.169 | 0.141 | 0.070 | 0.028 | 0.146 | 0.040 |
|  | d12 | 0.383 | 1.000 | 0.324 | 0.360 | 0.184 | 0.216 | 0.107 | 0.240 | 0.038 | 0.208 | 0.186 | 0.147 | 0.195 | 0.046 | 0.136 | 0.081 |
|  | c12 | 0.308 | 0.324 | 1.000 | 0.211 | 0.218 | 0.145 | 0.391 | 0.312 | 0.102 | 0.076 | 0.403 | 0.077 | 0.137 | 0.059 | 0.306 | 0.169 |
|  | p12 | 0.271 | 0.360 | 0.211 | 1.000 | 0.122 | 0.186 | 0.119 | 0.276 | 0.011 | 0.075 | 0.143 | 0.229 | 0.048 | -0.014 | 0.048 | -0.034 |
|  | si18 | 0.288 | 0.184 | 0.218 | 0.122 | 1.000 | 0.276 | 0.128 | 0.298 | 0.068 | 0.092 | 0.169 | -0.002 | 0.148 | 0.077 | 0.150 | 0.151 |
|  | d18 | 0.140 | 0.216 | 0.145 | 0.186 | 0.276 | 1.000 | 0.305 | 0.419 | 0.014 | 0.034 | 0.086 | 0.062 | 0.007 | 0.074 | 0.140 | 0.126 |
|  | c18 | 0.149 | 0.107 | 0.391 | 0.119 | 0.128 | 0.305 | 1.000 | 0.332 | 0.101 | 0.086 | 0.215 | 0.057 | 0.038 | 0.081 | 0.402 | 0.119 |
|  | p18 | 0.290 | 0.240 | 0.312 | 0.276 | 0.298 | 0.419 | 0.332 | 1.000 | -0.002 | 0.073 | 0.113 | 0.109 | 0.041 | 0.089 | 0.217 | 0.141 |
| Younger twin | si12 | 0.147 | 0.038 | 0.102 | 0.011 | 0.068 | 0.014 | 0.101 | -0.002 | 1.000 | 0.407 | 0.189 | 0.101 | 0.156 | 0.082 | 0.085 | 0.161 |
|  | d12 | 0.149 | 0.208 | 0.076 | 0.075 | 0.092 | 0.034 | 0.086 | 0.073 | 0.407 | 1.000 | 0.271 | 0.037 | 0.299 | 0.144 | 0.115 | 0.150 |
|  | c12 | 0.169 | 0.186 | 0.403 | 0.143 | 0.169 | 0.086 | 0.215 | 0.113 | 0.189 | 0.271 | 1.000 | 0.124 | 0.188 | 0.095 | 0.413 | 0.132 |
|  | p12 | 0.141 | 0.147 | 0.077 | 0.229 | -0.002 | 0.062 | 0.057 | 0.109 | 0.101 | 0.037 | 0.124 | 1.000 | 0.059 | 0.020 | 0.002 | 0.103 |
|  | si18 | 0.070 | 0.195 | 0.137 | 0.048 | 0.148 | 0.007 | 0.038 | 0.041 | 0.156 | 0.299 | 0.188 | 0.059 | 1.000 | 0.166 | 0.238 | 0.114 |
|  | d18 | 0.028 | 0.046 | 0.059 | -0.014 | 0.077 | 0.074 | 0.081 | 0.089 | 0.082 | 0.144 | 0.095 | 0.020 | 0.166 | 1.000 | 0.227 | 0.281 |
|  | c18 | 0.146 | 0.136 | 0.306 | 0.048 | 0.150 | 0.140 | 0.402 | 0.217 | 0.085 | 0.115 | 0.413 | 0.002 | 0.238 | 0.227 | 1.000 | 0.230 |
|  | p18 | 0.040 | 0.081 | 0.169 | -0.034 | 0.151 | 0.126 | 0.119 | 0.141 | 0.161 | 0.150 | 0.132 | 0.103 | 0.114 | 0.281 | 0.230 | 1.000 |

### Means and standard deviations for all variables

| **Table S13.** Trait means and standard deviations for MZ twins | | | | |
| --- | --- | --- | --- | --- |
|  | **n** | **Mean** | **Standard Deviation** | **Standard Error** |
| Isolation 12 | 596 | 0.88 | 1.33 | 0.05 |
| Anxiety 12 | 591 | 7.52 | 3.01 | 0.12 |
| Depression 12 | 592 | 3.06 | 5.38 | 0.22 |
| Conduct problems 12 | 587 | 2.47 | 2.98 | 0.12 |
| Psychosis 12 | 590 | 0.08 | 0.39 | 0.02 |
| Isolation 18 | 580 | 3.34 | 4.43 | 0.18 |
| Anxiety 18 | 579 | 0.98 | 1.84 | 0.08 |
| Depression 18 | 581 | 1.76 | 2.93 | 0.12 |
| Conduct problems 18 | 577 | 2.10 | 2.34 | 0.10 |
| Psychosis 18 | 581 | 1.31 | 2.73 | 0.11 |

| **Table S14.** Trait means and standard deviations for DZ twins | | | | |
| --- | --- | --- | --- | --- |
|  | **n** | **Mean** | **Standard Deviation** | **Standard Error** |
| Isolation 12 | 477 | 1.11 | 1.50 | 0.07 |
| Anxiety 12 | 474 | 7.73 | 3.14 | 0.14 |
| Depression 12 | 474 | 3.05 | 5.30 | 0.24 |
| Conduct problems 12 | 473 | 2.52 | 3.14 | 0.14 |
| Psychosis 12 | 473 | 0.11 | 0.45 | 0.02 |
| Isolation 18 | 448 | 3.45 | 4.58 | 0.22 |
| Anxiety 18 | 447 | 1.02 | 1.89 | 0.09 |
| Depression 18 | 449 | 1.81 | 3.01 | 0.14 |
| Conduct problems 18 | 447 | 2.23 | 2.34 | 0.11 |
| Psychosis 18 | 449 | 1.24 | 2.68 | 0.13 |

###

### Unconstrained independent pathway model

**Figure S3** shows the unconstrained independent pathway model where all paths have been freely estimated and different paths by sex have been specified for conduct problems. Very small non-significant loadings, or those that were estimated to be negative, were constrained to be zero which is shown in the main text. Constrained paths include: A_C1_ for conduct18, A_C2_ for psychosis18, C_C1_ for psychosis12, C_C2_ for isolation18, A_S2_ for depression12, A_S3_ for conduct12 males, A_S7_ for conduct18, C_S1_ for isolation12, C_S3_ for conduct12 females, C_S5_ for isolation18, C_S6_ for depression18, C_S7_ for conduct18, and C_S8_ for psychosis18.


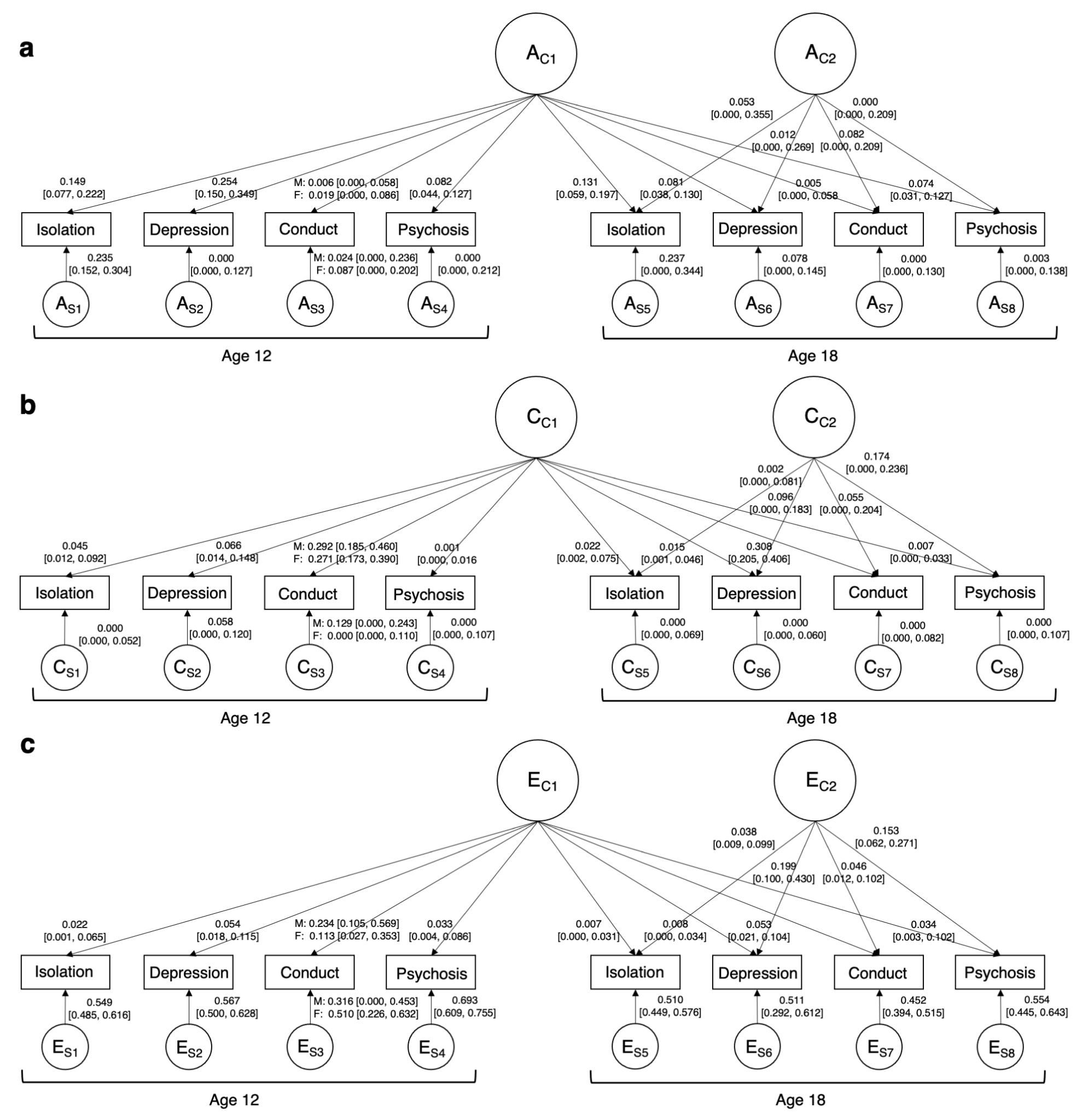


**Figure S3.** Independent pathway model (IPM) across ages 12 to 18. *A =* additive genetic influences, *C* = shared environmental influences, *E* = non-shared environmental influences on social isolation, depression symptoms, conduct problems, and psychotic experiences. Subscript C1 denotes common influences shared between traits and across time. Subscript C2 denotes common influences shared between traits at age 18. Subscript S1-8 denotes time- and trait-specific, residual influences. Solid paths denote A, C, and E influences on the variables at ages 12 and 18; the numbers on each path indicate their standardised contributions and their 95% confidence intervals.

###

### Female only contributions to the independent pathway model

As separate paths were specified for males and females for **conduct problems at age 12**, the contribution of ACE to the association between social isolation and conduct problems will be slightly different for males and females. See **Figure S4** for the female only graph of ACE contributions.


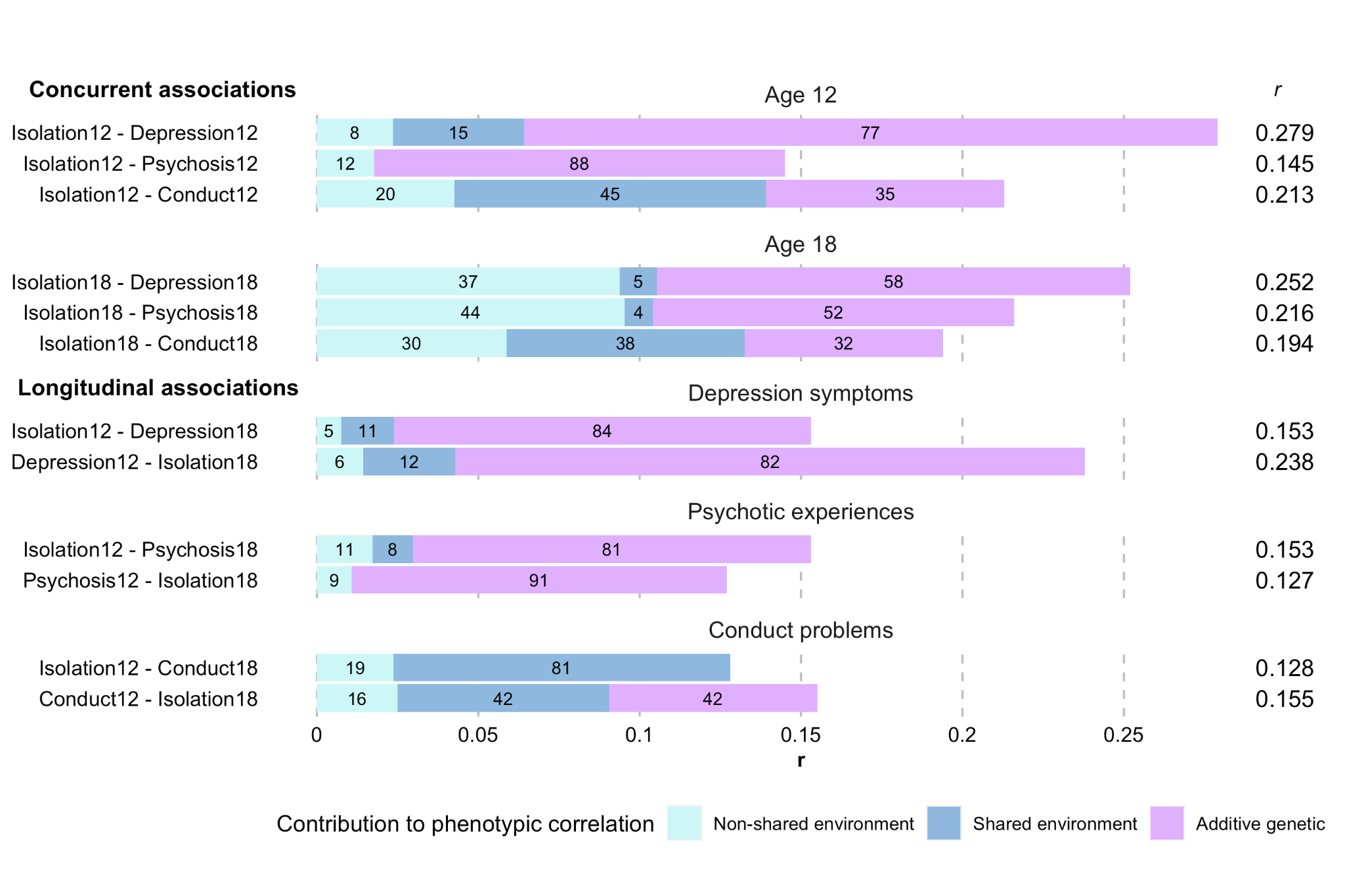


**Figure S4**. Proportions of concurrent and longitudinal associations between social isolation and mental health problems that are due to additive genetic, shared environmental, and non-shared environmental influences in male participants only. Estimated correlation coefficients (r) provided on the right-hand side of the bars.

Heritability was low for males (2%) and common across other traits. For females, heritability was higher (16%) and specific to conduct problems only. At age 18 these sex differences disappeared and genetic influences on conduct problems were common across all traits. Shared environmental influences on conduct problems at age 12 also differed by sex. For boys, 40% were specific to conduct problems, whereas for girls, 100% contributed to those common across other traits. Conversely, influences from the non-shared environment were more specific for girls, and common for boys. Uncovering genetic factors for conduct disorder may require attention to aetiological developmental changes (Pingault et al., 2015), improved granularity in measurement across time (Viding & McCrory, 2020), or considering a broader externalising spectrum (Beauchaine et al., 2017).

## Part D. Post hoc sensitivity analysis for the association between social isolation and conduct problems

We conducted a post hoc sensitivity analysis to unpick the large contribution of shared environmental influences between social isolation and conduct problems. To assess if the combined reporter score was influencing the C component in the association between social isolation (age 12) and conduct disorder (age 12 and 18) we computed two psychometric bivariate twin models. Social isolation at age 12 was assessed using a mother and teacher reported scale and conduct problems was assessed using a self-report measure based on the DSM-IV criteria for conduct disorder. Assessments for all other variables at all other ages were self-reported. Therefore, we incorporated a psychometric factor that captures social isolation that is common across mothers and teachers at age 12. We applied this psychometric structure for social isolation to two bivariate models with conduct problems at age 12 and 18 (**Figure S5**). As the factor has only two indicators (mother and teacher report), we applied the following SEM constraints to identify the model: specified equal residual ACE effects across mother and teacher reports, scaled the factor loadings for mother and teacher report to the first loading (mother reports), fixed the loading for conduct problems to be one and the ACE residuals to zero (to represent the same variance components as the observed variable).


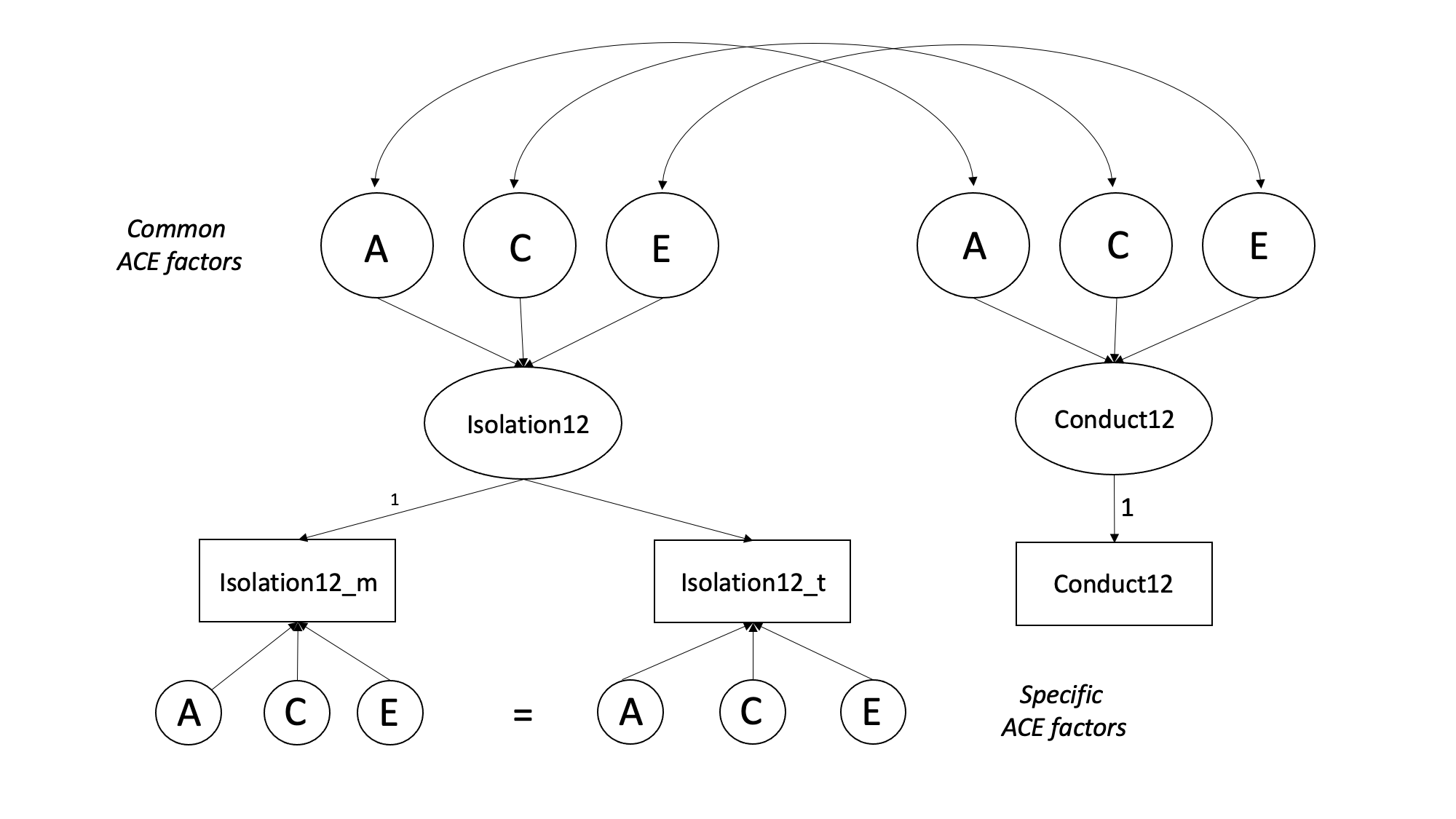


**Figure S5**. Psychometric bivariate ACE model for social isolation and conduct problems at age 12.

Social isolation heritability, shared environment, and unique environment estimates were very similar across reporters (**Table S15**). The psychometric specification did not lead to a significant loss in fit compared to a standard trivariate correlated factors specification (**Table S16**, **Table S17**). Mother and teacher reports of social isolation at age 12 loaded equally onto the isolation factor (factor loading = 0.31). The heritability of the common factor (~60%) was higher than that of the combined sum score (~41%), and the reporters separately (~38%; **Table S15**). The majority of the reporter-specific variance was attributable to the unique environment, whereas the majority of the reporter-common variance was attributable to additive genetic influences (**Figure S6**, **Table S18**). When mothers and teachers agree on the level of social isolation, ~60% was attributable to genetic influences and ~40% to the unique environment. Therefore, genetic factors play a large role in social isolation that is persistent across home and school contexts. When mothers and teachers disagree on levels of social isolation, genetic influences contribute to a lesser extent. The unique environment largely contributed to reporter-specific situational social isolation which captures both context-specific environmental variance and random error. Whilst the common social isolation factor represents a bias-free estimate of genetic and environmental influences on social isolation, a focus on reporter agreement could be incomplete due to the absence of rater-specific additive genetic variance which captures the unique perspective of mothers and teachers on social isolation. Further research is needed across different reporters and contexts to separate true situational influences on social isolation from measurement error.

The common social isolation factor resulted in slightly different proportions of genetic, common environment, and unique environment to the phenotypic correlations (**Table S19**, **Table S20**). At both ages, the genetic contribution to the phenotypic correlation was much larger than that found in the independent pathway model (IPM; **Table S20**). This aligns with the higher heritability found for the common social isolation factor, as well as the higher heritability for conduct problems that was captured by other traits in the IPM.

Therefore, our sensitivity analysis showed that considering the role of the reporter and the context in which social behaviour occurs is important in understanding this genetic and environmental overlap. The heritability of social isolation increased (~60%) when modelling agreement across mothers and teachers. The majority of the reporter-specific variance was attributable to the unique environment, whereas the majority of the reporter-common variance was attributable to additive genetic influences. This subsequently increased the contribution of additive genetic influences to the association between social isolation and conduct problems (At age 12: 62% due to A and 20% due to C; At age 18: 55% due to A and 45% due to C). When mothers and teachers agree on experiences of social isolation, thus capturing context-consistent isolation, genetic influences play a larger role in the association with conduct problems. This emphasises that different sources of heterogeneity should be considered in longitudinal genetically sensitive study designs.

| **Table S15**. Heritability, shared environment, and unique environment estimates for social isolation and conduct problems. | | | | | | |
| --- | --- | --- | --- | --- | --- | --- |
|  | **Isolation 12 summed reports** | **Isolation 12 mother report** | **Isolation 12 teacher report** | **Isolation 12 factor** | **Conduct 12** | **Conduct 18** |
| **h2** | 41.08% | 38.86% | 38.09% | 61.50% | 11.66% | 18.33% |
| **c2** | 00.64% | 00.02% | 00.08% | 01.10% | 32.83% | 27.88% |
| **e2** | 58.27% | 60.92% | 61.09% | 37.40% | 55.49% | 53.78% |
| Note. Estimates provided from several multivariate ACE models between social isolation and conduct problems. | | | | | | |

| **Table S16**. Comparison of fit across psychometric and correlated factors solutions age 12 conduct problems | | | | | | | | |
| --- | --- | --- | --- | --- | --- | --- | --- | --- |
| **Model** | **Comparison** | **ep** | **minus2LL** | **df** | **AIC** | **diffLL** | **diffdf** | **p** |
| Corfact | - | 36 | 16386.72 | 6000 | 16458.72 | - | - | - |
| Corfact | Psychometric | 16 | 16386.72 | 6020 | 16435.56 | 16.84189 | 20 | 0.6632151 |

| **Table S17**. Comparison of fit across psychometric and correlated factors solutions age 18 conduct problems | | | | | | | | |
| --- | --- | --- | --- | --- | --- | --- | --- | --- |
| **Model** | **Comparison** | **ep** | **minus2LL** | **df** | **AIC** | **diffLL** | **diffdf** | **p** |
| Corfact | - | 36 | 16205.112 | 5933 | 16277.11 | - | - | - |
| Corfact | Psychometric | 16 | 16225.29 | 5933 | 16257.29 | 20.17636 | 20 | 0.4469474 |

| **Table S18**. Standardised ACE estimates for the psychometric model for social isolation and conduct problems at age 12 and 18. | | | |
| --- | --- | --- | --- |
|  | **Isolation 12 common factor** | **Isolation 12 specific*** | **Conduct** |
| **Age 12 conduct** | | | |
| A | 0.615 [0.400, 0.776] | 0.196 [0.129, 0.256] | 0.118 [0.001, 0.313] |
| C | 0.011 [0.000, 0.157] | 0.000 [0.000, 0.038] | 0.327 [0.155, 0.446] |
| E | 0.374 [0.218, 0.540] | 0.491 [0.433, 0.557] | 0.555 [0.498, 0.616] |
| **Age 18 conduct problems** | | | |
| A | 0.592 [0.361, 0.761] | 0.196 [0.129, 0.256] | 0.182 [0.003, 0.379] |
| C | 0.023 [0.000, 0.184] | 0.000 [0.000, 0.000] | 0.282 [0.106, 0.439] |
| E | 0.385 [0.223, 0.552] | 0.491 [0.432, 0.556] | 0.536 [0.479, 0.600] |
| *Unstandardised effects were set to be equal across mother and teacher reports. | | | |

| **Table S19**. Correlations between social isolation and conduct problems factors. | | | |
| --- | --- | --- | --- |
|  | **Age 12 conduct** | **Age 18 conduct** | **Age 18 conduct*** |
| **rPh** | 0.291 | 0.192 | 0.196 |
| **rA** | 0.678 [0.043, 1.000] | 0.380 [-0.200, 1.000] | 0.320 [-0.155, 1.000] |
| **rC** | 1.000 [0.000, 1.000] | 1.000 [-1.000, 1.000] | 1.000 [-0.274, 1.000] |
| **rE** | 0.107 [-0.037, 0.252] | -0.026 [-0.174, 0.119] | 0* |
| * rE set to zero. | | |  |

| **Table S20**. Proportion of the phenotypic correlation due to ACE influences | | | |
| --- | --- | --- | --- |
|  | **A** | **C** | **E** |
| **Age 12 (rPh = 0.291)** | 62.59% | 20.57% | 16.83% |
| **Age 18 (rPh = 0.192)** | 64.68% | 41.62% | -6.30% |
| **Age 18* r(Ph = 0.196)** | 55.05% | 44.95% | 0%* |
| * rE set to zero. | | | |


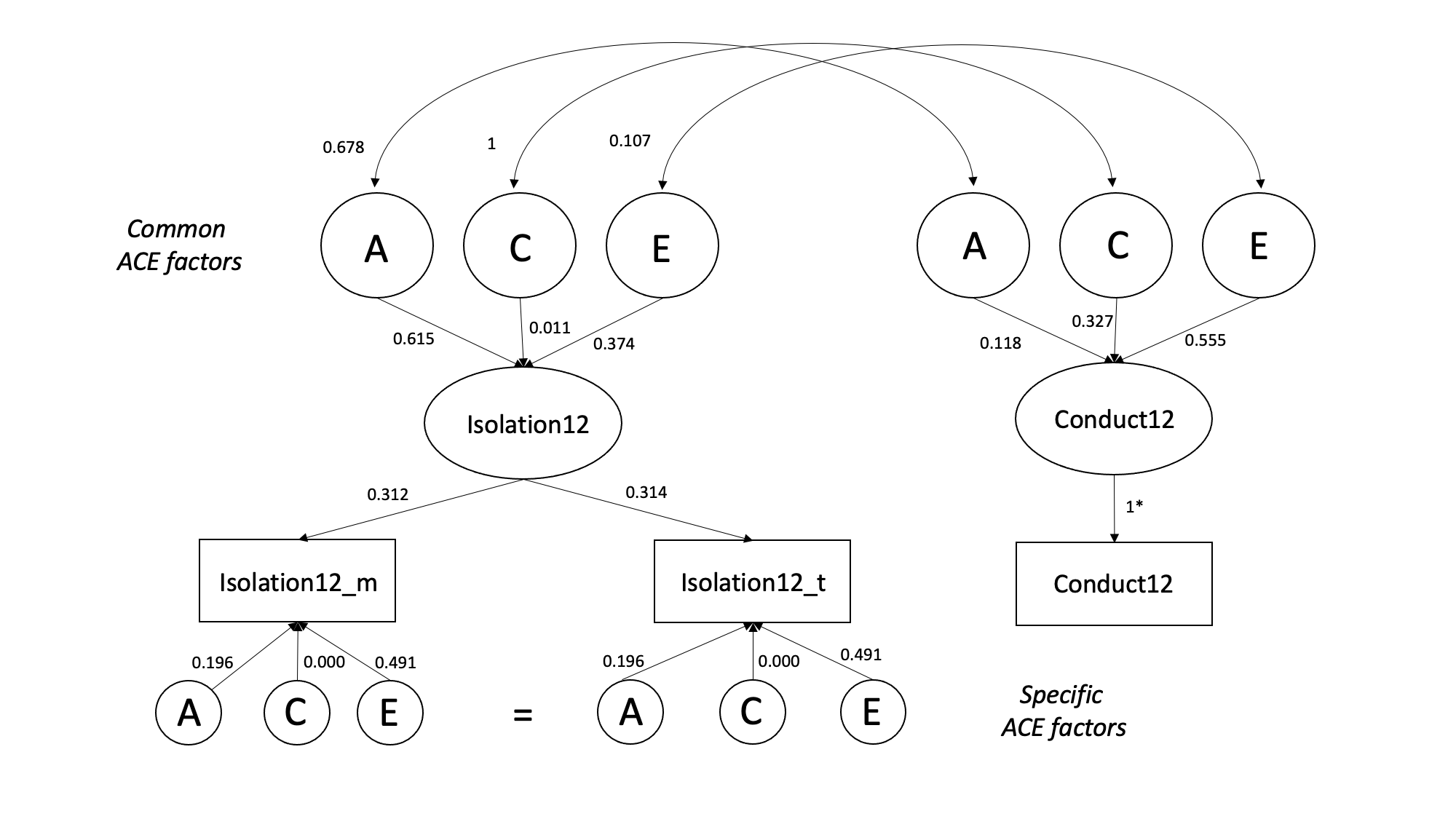


**Figure S6**. Psychometric bivariate twin model for social isolation and conduct problems at age 12.

# Supplement 4: R functions to replicate independent pathway model

We created two R functions to compute the IPM shown in this paper. First, **IPM_ace()** computes longitudinal independent pathway models at two time points without needing to specify the full twin modelling code. This function is found under the ipm_functions.R script on GitHub. We also provide an option to specify scalars in your model using the function **IPM_ace_scalar()**. Second, **IPM_ace_estimates_8var()** can be used to calculate the contribution proportions for ACE across a model using 8 variables. This function can be adapted for a different number of variables.

Link to the GitHub: <https://github.com/knthompson26/isolation_mentalhealth_overlap>

#

#

# References

Achenbach, T. M. (1991). *Manual for the Teacher’s Report Form and 1991 profile*. University of Vermont, Department of Psychiatry.

Achenbach, T. M., & Edelbrock, C. (1991). *Child behavior checklist* (Vol. 7, pp. 371–391). Burlington (Vt).

American Psychiatric Association. (1994). *Diagnostic and Statistical Manual of Mental Disorders, 4th edn*. Washington, DC: American Psychiatric Association.

Beauchaine, T. P., Zisner, A. R., & Sauder, C. L. (2017). Trait Impulsivity and the Externalizing Spectrum. *Annual Review of Clinical Psychology*, *13*(1), 343–368. https://doi.org/10.1146/annurev-clinpsy-021815-093253

Caspi, A., Harrington, H., Moffitt, T. E., Milne, B. J., & Poulton, R. (2006). Socially Isolated Children 20 Years Later: Risk of Cardiovascular Disease. *Archives of Pediatrics & Adolescent Medicine*, *160*(8), 805. https://doi.org/10.1001/archpedi.160.8.805

Kovacs, M. (1985). *The Children’s Depression Inventory (CDI)*. *21*, 995–998.

Loehlin, J. C. (1996). The Cholesky approach: A cautionary note. *Behavior Genetics*, *26*(1), 65–69. https://doi.org/10.1007/BF02361160

Matthews, T., Danese, A., Wertz, J., Ambler, A., Kelly, M., Diver, A., Caspi, A., Moffitt, T. E., & Arseneault, L. (2015). Social Isolation and Mental Health at Primary and Secondary School Entry: A Longitudinal Cohort Study. *Journal of the American Academy of Child & Adolescent Psychiatry*, *54*(3), 225–232. https://doi.org/10.1016/j.jaac.2014.12.008

Matthews, T., Danese, A., Wertz, J., Odgers, C. L., Ambler, A., Moffitt, T. E., & Arseneault, L. (2016). Social isolation, loneliness and depression in young adulthood: A behavioural genetic analysis. *Social Psychiatry and Psychiatric Epidemiology*, *51*(3), 339–348. https://doi.org/10.1007/s00127-016-1178-7

Newbury, J. B., Arseneault, L., Moffitt, T. E., Odgers, C. L., Howe, L. D., Bakolis, I., Reuben, A., Danese, A., Sugden, K., Williams, B., Rasmussen, L. J. H., Trotta, A., Ambler, A. P., & Fisher, H. L. (2023). Socioenvironmental Adversity and Adolescent Psychotic Experiences: Exploring Potential Mechanisms in a UK Longitudinal Cohort. *Schizophrenia Bulletin*, sbad017. https://doi.org/10.1093/schbul/sbad017

Pingault, J.-B., Rijsdijk, F., Zheng, Y., Plomin, R., & Viding, E. (2015). Developmentally dynamic genome: Evidence of genetic influences on increases and decreases in conduct problems from early childhood to adolescence. *Scientific Reports*, *5*(1), 10053. https://doi.org/10.1038/srep10053

Polanczyk, G., Moffitt, T. E., Arseneault, L., Cannon, M., Ambler, A., Keefe, R. S. E., Houts, R., Odgers, C. L., & Caspi, A. (2010). Etiological and Clinical Features of Childhood Psychotic Symptoms: Results From a Birth Cohort. *Archives of General Psychiatry*, *67*(4), 328. https://doi.org/10.1001/archgenpsychiatry.2010.14

Reuben, A., Sugden, K., Arseneault, L., Corcoran, D. L., Danese, A., Fisher, H. L., Moffitt, T. E., Newbury, J. B., Odgers, C., Prinz, J., Rasmussen, L. J. H., Williams, B., Mill, J., & Caspi, A. (2020). Association of Neighborhood Disadvantage in Childhood With DNA Methylation in Young Adulthood. *JAMA Network Open*, *3*(6), e206095–e206095. https://doi.org/10.1001/jamanetworkopen.2020.6095

Robins, L., Cottler, L., Bucholz, K., & Compton, W. (1995). *Diagnostic Interview Schedule for DSM-IV*.

Thompson, K. N., Agnew-Blais, J. C., Allegrini, A. G., Bryan, B. T., Danese, A., Odgers, C. L., Matthews, T., & Arseneault, L. (2023). Do Children With Attention-Deficit/Hyperactivity Disorder Symptoms Become Socially Isolated? Longitudinal Within-Person Associations in a Nationally Representative Cohort. *JAACAP Open*, *1*(1), 12–23. https://doi.org/10.1016/j.jaacop.2023.02.001

Thompson, K. N., Odgers, C. L., Bryan, B. T., Danese, A., Milne, B. J., Strange, L., Matthews, T., & Arseneault, L. (2022). Trajectories of childhood social isolation in a nationally representative cohort: Associations with antecedents and early adulthood outcomes. *JCPP Advances*, *2*(2). https://doi.org/10.1002/jcv2.12073

Viding, E., & McCrory, E. (2020). Disruptive Behavior Disorders: The Challenge of Delineating Mechanisms in the Face of Heterogeneity. *American Journal of Psychiatry*, *177*(9), 811–817. https://doi.org/10.1176/appi.ajp.2020.20070998

Zimet, G. D., Dahlem, N. W., Zimet, S. G., & Farley, G. K. (1988). The Multidimensional Scale of Perceived Social Support. *Journal of Personality Assessment*, *52*(1), 30–41. https://doi.org/10.1207/s15327752jpa5201_2
